# EMGMDA: a multi-modal graph neural framework for robust prediction of miRNA–disease associations

**DOI:** 10.1186/s12864-026-12834-4

**Published:** 2026-04-14

**Authors:** Jianan Sui, Deqiang Gu, Shichao Song, Xiaoqiang Shi, Zhenyu Cui

**Affiliations:** Department of Urology, Affiliated Hospital of Hebei University, Baoding, 071000 Hebei China

**Keywords:** miRNA-disease association, Histopathological image integration, Residual GraphSAGE, Multi-modal feature fusion, Triplet contrastive learning

## Abstract

Despite significant progress in miRNA–disease association prediction, accurate inference remains hampered by the extreme sparsity of interaction networks, the heterogeneity of biological data modalities, and the redundancy introduced by simple feature concatenation. These limitations are especially pronounced in major cancers such as lung, breast, colorectal, gastric, and liver, where similarity-based methods often fail to capture critical pathological characteristics, underscoring the need to integrate histopathological images for richer disease representation. To address these challenges, we propose EMGMDA, which applies residual GraphSAGE to the sparse miRNA–disease graph to generate robust neighbor-aggregated embeddings, employs a nonlinear adaptive fusion module to learn high-order miRNA–disease feature interactions and eliminate redundancy, integrates multi-scale histopathological features from The Cancer Genome Atlas (TCGA) whole-slide images via a pretrained ResNet-18 and cross-attention mechanism to heighten sensitivity to tumor heterogeneity, and leverages triplet contrastive learning to refine the embedding space by drawing true associations closer and separating unrelated pairs, thereby improving discrimination in data-scarce scenarios. Experiments show that EMGMDA achieves an AUC of 0.9641 and an AUPRC of 0.9599 on HMDD v2.0, and further elevates performance to an AUC of 0.9742 and an AUPRC of 0.9719 on HMDD v3.2, significantly surpassing state-of-the-art methods. Case studies on lung, esophageal, breast, and colorectal cancers further validate its reliability and practical utility.

**Supplementary Information:**

The online version contains supplementary material available at 10.1186/s12864-026-12834-4.

## Introduction

As small non-coding RNA molecules, microRNAs (miRNAs) play an essential role in gene expression control at the post-transcriptional stage, primarily through binding to target mRNAs and causing either inhibition of translation or mRNA decay [1]. As key regulators, miRNAs influence multiple biological pathways, notably those governing cell division, specialization, apoptosis, and immune system regulation [2–4]. Dysregulation of miRNA expression has been increasingly linked to the development and progression of various diseases [5, 6]. Due to their disease-specific expression patterns, miRNAs are being explored as potential biomarkers and therapeutic targets. Although conventional experimental approaches [7, 8] can provide reliable and precise results, they usually require substantial financial resources and lengthy procedures, making them both expensive and time-consuming. As a result, computational methods for predicting and identifying novel miRNA-disease associations have garnered increasing attention in recent years.

Several authoritative reviews have systematically summarized MDA prediction from experimental evidence to computational modeling. Chen et al. provided an early comprehensive overview of miRNA–disease studies, related databases, and representative computational models, and discussed major challenges for future MDA inference [9]. More recently, Huang et al. published an updated review series that revisits experimental findings, summarizes updated databases and webservers with an emphasis on multi-source data fusion, proposes an updated taxonomy of MDA predictors (e.g., fusion vs. non-fusion paradigms) with analyses of trends and challenges across model families, and highlights the lack of standardized evaluation practices by recommending a feasible workflow for fair and systematic assessment of MDA models [10–12]. Following the taxonomy summarized in these reviews, we organize representative MDA prediction methods into scoring-function-based models, traditional machine learning approaches, representation-learning methods (including matrix factorization and deep models), and graph neural network–based frameworks.

Scoring functions (SFs) are commonly employed in the prediction of miRNA–disease associations (MDAs), with the goal of establishing reliable metrics to assess the interaction strength between miRNAs and diseases. For instance, Jiang et al. [13] introduced a scoring framework designed to estimate the likelihood of a miRNA’s association with particular disease phenotypes. By prioritizing high-scoring miRNAs, this approach generates testable hypotheses for experimental validation, thereby streamlining the discovery process and reducing both cost and effort. Similarly, Yan et al. [14] presented WBSMDA, a computational scoring model capable of identifying candidate miRNAs associated with complex diseases, including cases where no known related miRNAs exist.

Besides scoring-based strategies, traditional machine learning techniques have also been extensively applied to predict miRNA–disease associations. For example, Xu et al. [15] proposed the miRNA Target Gene Dysregulation Network (MTDN), an SVM-based framework that integrates miRNA and mRNA expression data with target prediction information to identify cancer-associated miRNAs. Chen et al. [16] developed RFMDA, a prediction model built upon the random forest algorithm. In subsequent work, the same group introduced RBMMMDA [17], which leverages restricted Boltzmann machines to infer diverse types of miRNA–disease interactions. Additionally, Wang et al. [18] presented LMTRDA, a logistic model tree–based method that combines multiple data sources and applies natural language processing to extract sequence-derived features of miRNAs, achieving high predictive performance across different cancer types.

Beyond manually designed scoring rules, representation-learning methods aim to infer latent factors directly from multi-source similarity and association data. Deep learning approaches have demonstrated significant potential in predicting miRNA–disease associations. Chen et al. [19] introduced DBNMDA, which leverages a deep-belief network with RBM-based pretraining on all miRNA–disease pairs and subsequent fine-tuning with labeled samples to infer potential associations. Ji et al. [20] proposed AEMDA, a computational method that combines disease semantic similarity, miRNA functional similarity, and heterogeneous interaction information within a deep autoencoder framework. This model predicts associations by evaluating reconstruction error and was shown to achieve higher accuracy compared to earlier techniques. In a similar direction, Liu et al. [21] developed DFELMDA, which integrates deep forest ensemble learning with autoencoder features to enhance prediction performance, especially across diverse cancer types. Xu et al. [22] introduced PMFMDA, a model based on probabilistic matrix factorization that incorporates multiple similarity measures and association data to infer miRNA–disease interactions. Furthermore, Cui et al. [23] applied reinforcement learning through a Q-learning algorithm to optimize the weighting of three constituent prediction models, resulting in improved predictive accuracy.

As an increasingly prominent branch of deep learning, graph neural networks (GNNs) have attracted growing interest in bioinformatics for representing complex relational data in structured graph form, offering promising applications in predicting miRNA–disease association [24–27]. Chu et al. [28] proposed MDA-GCNFTG, which predicts miRNA–disease associations using graph convolutional networks with graph sampling on feature and topology graphs to improve training efficiency and predictive accuracy across multiple prediction tasks. Tang et al. [29] introduced MMGCN, a multi-view and multi-channel attentive graph convolutional network that dynamically weights feature importance and has achieved state-of-the-art performance across multiple evaluation metrics. Li et al. [30] proposed GAEMDA, a model leveraging graph autoencoders to learn low-dimensional node embeddings from heterogeneous biological data, showing particularly strong results in cancer-related predictions. Ding et al. [31] presented VGAE-MDA, a variational graph autoencoder framework that integrates outputs from dual sub-networks to enhance predictive accuracy, as confirmed in case analyses. Wang et al. [32] developed MAGCN, which employs multi-channel attention mechanisms within a GCN architecture to improve association inference. Zhang et al. [33] designed AGAEMD, incorporating node-level attention into graph autoencoders for refined relationship prediction. Dong et al. [34] proposed MDformer, a model that fuses multi-source features with topological information to achieve robust performance across major disease types. Li et al. [35] constructed HHOMR, which utilizes higher-order moment representations and joint spatial-feature aggregation, attaining an AUC of 93.28% in cross-validation and outperforming existing methods. Lastly, Zhao et al. [36] introduced MotifMDA, integrating both high- and low-order graph structures with motif-aware learning to further improve prediction on standard benchmarks. Tian et al. [37] presented MGCNSS, which integrates multi-layer graph convolution on a heterogeneous network with a distance-based negative sample selection strategy to improve MDA prediction under both balanced and imbalanced settings.

Despite recent progress, predicting miRNA–disease associations remains challenging. Models that rely primarily on similarity-based features may fail to capture key disease phenotypes, especially in major cancers such as lung, breast, colorectal, gastric, and liver, motivating the incorporation of histopathological images as complementary phenotype evidence. Importantly, aberrant molecular regulatory programs are often reflected in measurable alterations of tissue architecture and the tumor microenvironment, which can be observed in routine H&E slides [38–40]. Moreover, prior studies [41, 42] have demonstrated that deep learning models can predict certain molecular alterations from histopathology (e.g., microsatellite instability prediction in gastrointestinal cancers and mutation prediction in lung cancer), indicating that morphological patterns may carry information predictive of latent molecular states rather than serving as purely descriptive features. Collectively, these findings provide a biological rationale for integrating histopathological image features as complementary disease phenotype cues in miRNA–disease association modeling. From a biological perspective, histopathological morphology provides an important phenotypic readout of disease states and can be accompanied by widespread transcriptomic and post-transcriptional alterations, including miRNA dysregulation. Nevertheless, morphological patterns are shaped by multiple interacting pathways, microenvironmental cues, and cell-type compositions, and therefore should be interpreted as phenotype-level correlates of underlying regulatory shifts rather than direct evidence that specific miRNAs causally drive particular histological features. Consistent with this view, we do not treat morphological signals as causal proof of miRNA regulation; instead, in this work we adopt an association-centric formulation and aim to prioritize candidate miRNA–disease associations for downstream experimental validation. While GCNs and GATs show promise, the sparsity of miRNA-disease networks limits their effectiveness in modeling complex associations. Moreover, simple feature concatenation can introduce redundancy, obscuring the unique contributions of each data type and failing to capture vital nonlinear relationships. Consequently, these models underperform in data-sparse scenarios, underscoring the need for more robust integration strategies and advanced methodologies to enhance predictive accuracy.

**Fig. 1:**
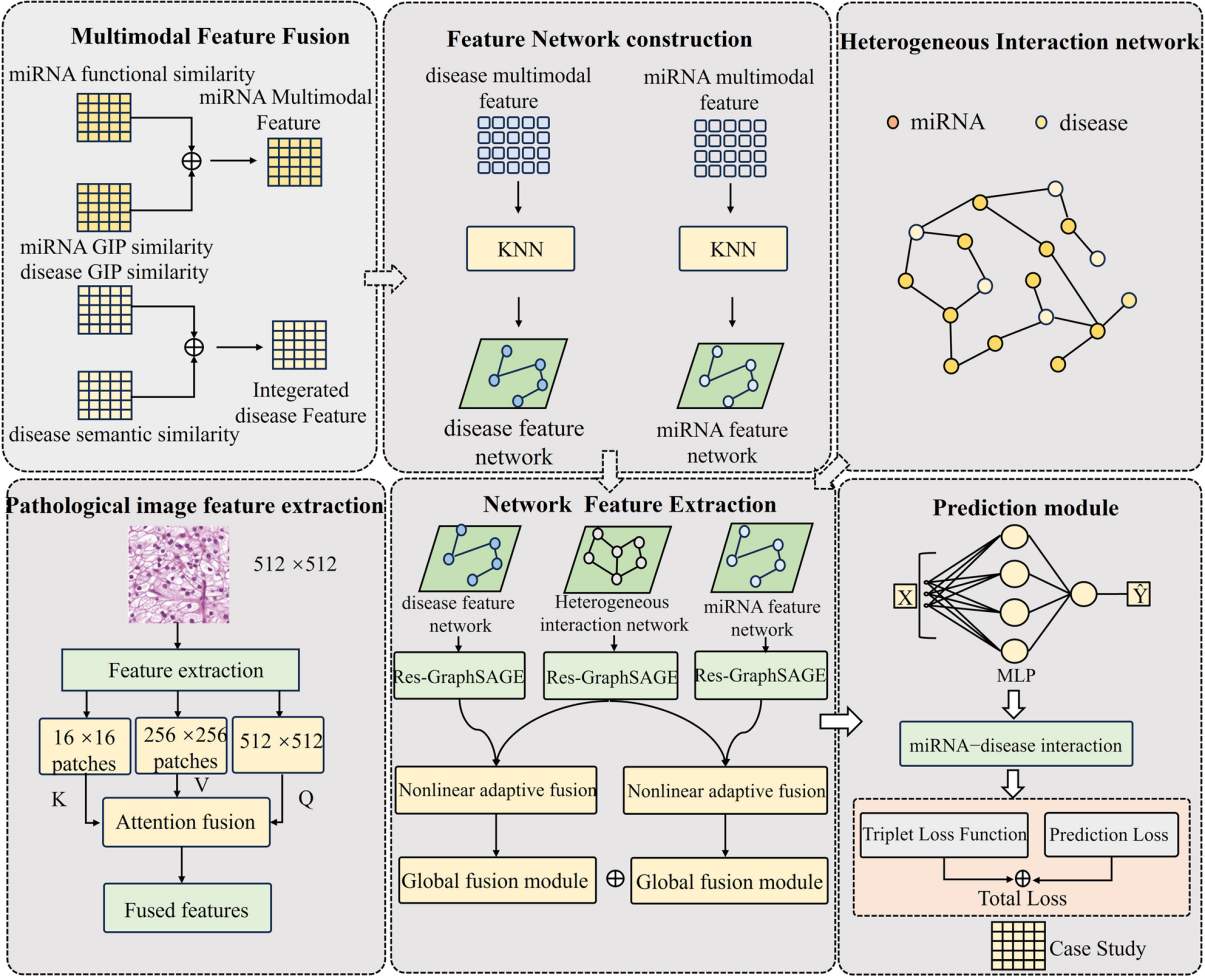
Flowchart of EMGMDA

To address these challenges, we introduce EMGMDA (Fig. 1), a novel framework for miRNA–disease association prediction that integrates multiple advanced methodologies. First, within the inherently sparse miRNA–disease interaction network, we adopt GraphSAGE to perform efficient neighbor sampling and feature aggregation, thereby yielding robust node embeddings. Residual connections are further employed to mitigate vanishing gradients and enhance both model capacity and training stability. In addition, EMGMDA employs a nonlinear adaptive fusion mechanism to capture higher-order miRNA–disease relationships, which helps reduce redundant information and enhances the utilization of diverse modalities beyond what simple concatenation can achieve. Specifically, SVS whole-slide images were downloaded from TCGA for five major cancer types (lung, breast, colorectal, gastric, and liver). For each cancer type, we sampled 300 patches per scale at three resolutions (512 × 512, 256 × 256, and 16 × 16), resulting in 900 patches per cancer type and 4,500 patches in total. Each patch was processed using a pretrained ResNet-18 backbone to obtain feature embeddings, and we fused multi-scale embeddings using a cross-attention module in which the 512-scale feature provides global context and adaptively aggregates complementary fine-scale cues from 256-scale and 16-scale features. Finally, we employ triplet contrastive learning to refine the embedding space by discriminating among miRNAs, their associated diseases, and unrelated diseases. This contrastive objective further strengthens the model’s capacity to distinguish true associations in data-scarce settings. Moreover, we augment our network data with histopathological image features.

## Materials and methods

### Datasets

**Fig. 2:**
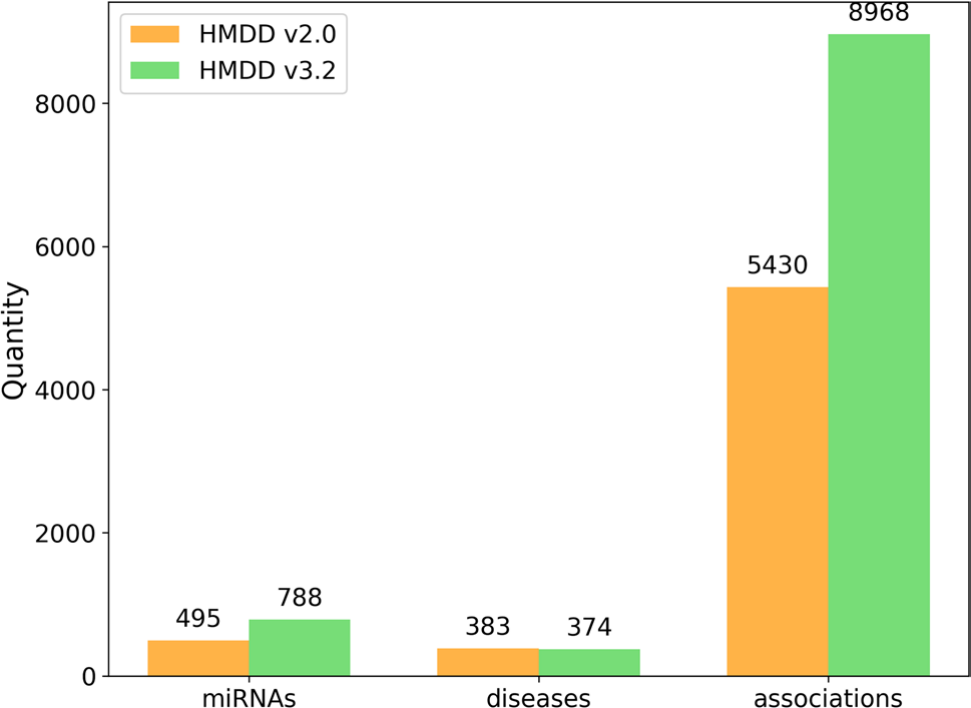
Basic statistics of the HMDD v2.0 and v3.2 datasets

The Human MicroRNA–Disease Database (HMDD) is one of the most authoritative and widely used repositories for experimentally validated miRNA–disease associations in the field of MDA prediction. To comprehensively evaluate both the effectiveness and generalization capability of our model, we employed two versions of HMDD: v2.0 [43] and v3.2 [44]. The basic statistics of these datasets are summarized in Fig. 2.From these datasets, we constructed a binary miRNA–disease association (MDA) matrix, in which each entry was assigned a value of 1 if a confirmed association between a given miRNA and disease was reported, and 0 otherwise. It is important to note that an entry of 0 does not indicate biological independence but rather reflects the absence of experimental evidence at the time.

### Collection and processing of morphology images

In this study, we downloaded SVS whole-slide images (WSIs) from The Cancer Genome Atlas (TCGA) [45] for five major cancer types, including lung, breast, colorectal, gastric, and liver cancers. For each cancer type, we constructed a three-scale patch dataset at 512 × 512, 256 × 256, and 16 × 16 pixels. Candidate patches were generated using a sliding-window strategy with overlap to ensure comprehensive coverage of diagnostically relevant regions and to reduce the risk of missing critical pathological patterns. From the candidate pool at each scale, we sampled 300 patches per cancer type, resulting in 900 patches per cancer type in total (300 at 512 × 512, 300 at 256 × 256, and 300 at 16 × 16), and 4,500 patches across the five cancer types. This multi-scale patch dataset provides a solid foundation for subsequent deep learning-based feature extraction and downstream predictive modeling.

### Similarity computation

#### Disease similarity network

To quantify semantic similarity between diseases, we employ the Medical Subject Headings (MeSH) ontology [46] and represent each disease as a directed acyclic graph (DAG). For a disease ddd, the graph is defined as

1 $$\:DAG\left(d\right)=\left(V\right(d),E(d\left)\right)$$

Here, $\:V\left(d\right)$ denotes the set of nodes associated with disease $\:d$, including $\:d$ itself and all of its ancestor terms in MeSH, while $\:E\left(d\right)$ represents the directed edges that capture parent–child relationships among these nodes.

The semantic weight of disease $\:d$, written as $\:{SV}_{1}\left(d\right)$, is calculated by:

The first semantic weight of disease $\:d$, denoted $\:{SV}_{1}\left(d\right)$, aggregates the contributions of all nodes in $\:V\left(d\right)$:

2 $$\:{SV}_{1}\left(d\right)=\sum\:_{u\in\:V\left(d\right)}{\phi\:}_{1}\left(d,u\right)\:$$

where $\:{\phi\:}_{1}\left(d,u\right)$ is the semantic contribution of node *u* to disease *d*, defined as: $$\:{\phi\:}_{1}(d,\mathrm{u})=\{\begin{array}{cc}1,&\:\text{}\mathrm{u}\text{}\mathrm{=}\text{}\mathrm{d}\\\:\mathrm{max}\left\{\lambda\:\bullet\:{\phi\:}_{1}\left(d,c\right)\mid\mathrm{c}\in\:\mathrm{children}\mathrm{(}\mathrm{u}\mathrm{)}\right\},&\;\mathrm{u}\ne\:\mathrm{d}\end{array}$$ (3).

Here, λ = 0.5 is a decay factor that regulates the influence transmitted through hierarchical levels. The first semantic similarity between two diseases $\:{d}_{i}$ and $\:{d}_{j}$ is then defined as:

4 $$\:\:\:\:\:\:{S}_{1}\left({d}_{i},{d}_{j}\right)=\frac{\sum\:_{k\in\:V\left({d}_{i}\right)\cap\:V\left({d}_{j}\right)}\left({\phi\:}_{1}\left({d}_{i},k\right)+{\phi\:}_{1}\left({d}_{j},k\right)\right)}{{SV}_{1}\left({d}_{i}\right)+{SV}_{1}\left({d}_{j}\right)}$$

To further capture hierarchical information, we introduce an alternative semantic weight based on information content:

5 $$\:\:\:{SV}_{2}\left(d\right)=\sum\:_{u\in\:V\left(d\right)}-\mathrm{log}\left(\frac{f\left(u\right)}{\left|V\right|}\right)$$

where $\:\left|V\right|$denotes the total number of nodes across all DAGs, and $\:f\left(u\right)$ represents the frequency of occurrence of node $\:u$. Based on this definition, the second semantic similarity between diseases $\:{d}_{i}$ and$\:{d}_{j}$ is formulated as:

6 $$\:{S}_{2}\left({d}_{i},{d}_{j}\right)=\frac{\sum\:_{k\in\:V\left({d}_{i}\right)\cap\:V\left({d}_{j}\right)}\left({\phi\:}_{2}\left({d}_{i},k\right)+{\phi\:}_{2}\left({d}_{j},k\right)\right)}{{SV}_{2}\left({d}_{i}\right)+{SV}_{2}\left({d}_{j}\right)}$$

Finally, the integrated semantic similarity is computed as the mean of the two measures:

7 $$\:{S}_{D}\left({d}_{i},{d}_{j}\right)=\frac{{S}_{1}\left({d}_{i},{d}_{j}\right)+{S}_{2}\left({d}_{i},{d}_{j}\right)}{2}$$

#### miRNA functional similarity

The functional similarity among miRNAs is assessed using the method introduced by Wang et al. [47]. For two miRNAs $\:{m}_{i}$ and $\:{m}_{j}$, the functional similarity is given by:

8 $$\:FS\left({m}_{i},{m}_{j}\right)=\frac{\sum\:_{{d}_{i}\in\:D{M}_{i}}Sim\left({d}_{i},D{M}_{j}\right)}{p+q}+\frac{\sum\:_{{d}_{j}\in\:D{M}_{j}}Sim\left({d}_{j},D{M}_{i}\right)}{p+q}$$

Here, $\:D{M}_{i}$ and $\:D{M}_{j}$ refer to the collections of diseases associated with $\:{m}_{i}$ and $\:{m}_{j}$, respectively, where $\:p$ and $\:q$ denote the number of elements in each set.

For a disease $\:{d}_{k}$, its maximum similarity with diseases in set $\:DM$ is computed as:

9 $$\:\begin{array}{c}Sim\left({d}_{t},DM\right)=\underset{{d}_{i}\in\:DM}{max}\:\left(Sim\left({d}_{k},{d}_{i}\right)\right)\end{array}$$

This framework quantifies the functional similarity of miRNAs by considering their disease contexts, thereby enhancing insights into the roles of miRNAs in disease progression.

#### Gaussian interaction profile kernel similarity

To mitigate sparsity in similarity measures, we adopt the Gaussian Interaction Profile (GIP) kernel [20]. For an miRNA $\:{m}_{i}$, its interaction profile $\:IP\left({m}_{i}\right)$ corresponds to the *i*-th row of the miRNA–disease association matrix, and the similarity between two miRNAs is:

10 $$\:{K}_{m}\left({m}_{i},{m}_{j}\right)=\mathrm{exp}\left(-{\eta\:}_{m}\cdot\:\parallel\:IP\left({m}_{i}\right)-IP\left({m}_{j}\right){\parallel\:}^{2}\right)$$

where the kernel bandwidth parameter $\:{\eta\:}_{m}$ is given by:

11 $$\:{\eta\:}_{m}=\frac{{\gamma\:}_{m}^{{\prime\:}}}{\frac{1}{M}\sum\:_{i=1}^{M}\:\parallel\:IP\left({m}_{i}\right){\parallel\:}^{2}}$$

Following previous work [48], $\:{\gamma\:}_{m}^{{\prime\:}}$ is set to 1, where *M* is the number of miRNAs, $\:IP\left({m}_{i}\right)$ is the interaction profile of miRNA $\:{m}_{i}$ (a binary vector indicating its disease associations), and ∥⋅∥ denotes the Euclidean norm.

For diseases, the kernel is defined analogously:

12 $$\:{K}_{d}\left({d}_{i},{d}_{j}\right)=\mathrm{exp}\left(-{\eta\:}_{d}\cdot\:\parallel\:IP\left({d}_{i}\right)-IP\left({d}_{j}\right){\parallel\:}^{2}\right)$$

13 $$\:{\eta\:}_{d}=\frac{{\gamma\:}_{d}^{{\prime\:}}}{\frac{1}{D}\sum\:_{i=1}^{D}\:\parallel\:IP\left({d}_{i}\right){\parallel\:}^{2}}$$

Where *D* is the number of diseases, and $\:IP\left({d}_{i}\right)$ is the interaction profile of disease $\:{d}_{i}$, i.e., a binary vector showing its associations with miRNAs. This kernel effectively captures similarity among both miRNAs and diseases by directly comparing their interaction patterns.

This method thus provides an efficient kernel-based tool for quantifying similarities among both miRNAs and diseases.

#### Integrated similarity

To produce more robust similarity matrices, we integrate the semantic/functional similarities with the GIP kernel similarities. Specifically, the integrated disease similarity is:

14 $$\:I{S}_{D}\left({d}_{i},{d}_{j}\right)=\left\{\begin{array}{c}{S}_{D}\left({d}_{i},{d}_{j}\right)\:\:\:\:\:\:\:\:\:\:\:\:\:\:\:{S}_{D}\left({d}_{i},{d}_{j}\right)>0\\\:{K}_{d}\left({d}_{i},{d}_{j}\right)\:\:\:\:\:\:\:\:\:\:\:\:\:\:{S}_{D}({d}_{i},{d}_{j})=0\end{array}\right.$$

Similarly, the integrated miRNA similarity is defined as:

15 $$\:I{S}_{M}\left({m}_{i},{m}_{j}\right)=\left\{\begin{array}{c}FS\left({m}_{i},{m}_{j}\right)\:\:\:\:\:\:\:\:\:\:\:\:\:\:\:FS\left({m}_{i},{m}_{j}\right)>0\\\:{K}_{m}\left({m}_{i},{m}_{j}\right)\:\:\:\:\:\:\:\:\:\:\:\:\:\:FS({m}_{i},{m}_{j})=0\end{array}\right.$$

This hybrid design leverages both ontology-based measures and kernel-based profiles, ensuring reliable similarity quantification even in sparse association scenarios.

### Proposed model

#### Graph construction and initial node features

To ensure reproducibility and methodological clarity, we explicitly describe the construction of graph structures and the initialization of node features for all graphs used in this study, including the miRNA similarity graph, the disease similarity graph, and the miRNA–disease association graph.

The initial node features are defined according to the characteristics of each graph. For the miRNA similarity graph, each miRNA node is initialized with its functional similarity interaction profile, corresponding to the associated row of the miRNA functional similarity matrix. For the disease similarity graph, each disease node is initialized using its semantic similarity interaction profile obtained from the integrated disease similarity matrix described in Sect. (Similarity computation). For the miRNA–disease association graph, node features are constructed from interaction profiles derived from known miRNA–disease associations: miRNA nodes use their miRNA–disease association vectors, while disease nodes use the corresponding transposed vectors. These interaction profiles are subsequently projected into a shared latent space through linear transformations before being fed into the graph convolutional layers.

For the miRNA similarity graph and the disease similarity graph, adjacency matrices are constructed using a K-nearest neighbor (KNN) strategy based on the integrated similarity matrices defined in Sect. (Similarity computation). Specifically, for each node $\:{v}_{i}$, its top-$\:K$ most similar nodes are selected to form the neighbor set $\:{\mathcal{N}}_{K}\left({v}_{i}\right)$. An undirected edge is established if either node appears in the other’s KNN set, and self-loops are retained to preserve node-specific information. In all experiments, the number of nearest neighbors is set to $\:K=20$. The adjacency matrix is defined as:

16 $$\:{A}_{ij}=\left\{\begin{array}{c}1,\:\:\:\:\:\:\:\:\:\:\:\:\:\:\:\:\:\:\:\:{v}_{j}\in\:Nk\left({v}_{i}\right)\\\:0,\:\:\:\:\:\:\:\:\:\:\:\:\:\:\:\:\:\:\:\:\:\mathrm{otherwise}\end{array}\right.$$

For the miRNA–disease association graph, no KNN construction is performed. Instead, the known bipartite adjacency matrix derived from experimentally validated miRNA–disease associations is directly used to define graph connectivity.

#### GraphSAGE propagation and aggregation

We adopt a three-layer GraphSAGE architecture to learn node embeddings from each graph, employing mean aggregation at every layer. For a node $\:v$, the neighborhood aggregation at the $\:l$-th layer is defined as:

17 $$\:{h}_{\mathcal{N}\left(v\right)}^{\left(l\right)}=\frac{1}{\mid\:\mathcal{N}\left(v\right)\mid\:}\sum\:_{u\in\:\mathcal{N}\left(v\right)}{h}_{u}^{\left(l\right)}$$

The node representation is then updated by concatenating its own embedding with the aggregated neighborhood information:

18 $$\:{h}_{v}^{\left(l+1\right)}=\sigma\:\left({W}^{\left(l\right)}\cdot\left[{h}_{v}^{\left(l\right)}\hspace{0.17em}\parallel\:\hspace{0.17em}{h}_{\mathcal{N}\left(v\right)}^{\left(l\right)}\right]\right)$$

where $\:{W}^{\left(\boldsymbol{l}\right)}$denotes a trainable weight matrix, [⋅∥⋅] represents concatenation, and $\:\sigma\:(\cdot\:)$is the ELU activation function.

The hidden dimensions of the three GraphSAGE layers are set to 128, 64, and $\:d$, respectively.

#### Residual connection design

To alleviate gradient vanishing and representation degradation in deeper graph convolutional networks, we incorporate linear residual connections inspired by ResNet. The residual update rule is formulated as:

19 $$\:{\stackrel{\sim}{h}}_{v}^{\left(l+1\right)}=\mathrm{ELU}\left({h}_{v}^{\left(l+1\right)}\right)+{W}_{r}{h}_{v}^{\left(l\right)}$$

where $\:{W}_{r}$ is a trainable linear projection matrix used to align feature dimensions across successive layers.

This residual mechanism enables the learned node embeddings to preserve both original node semantics and multi-hop neighborhood information, resulting in more stable and expressive representations.

#### Nonlinear adaptive fusion of feature embeddings

To integrate complementary information learned from different graph views, we introduce a nonlinear adaptive fusion module to combine two embeddings obtained for the same entity type (miRNA or disease) from different graphs/branches. In our framework, each input embedding matrix is produced by the graph representation learning process (i.e., after graph propagation/aggregation). Let $\:{X}_{m}^{\left(1\right)}$and $\:{X}_{m}^{\left(2\right)}$ denote two miRNA-view embedding matrices, which are obtained from the GraphSAGE output on the miRNA–miRNA similarity graph $\:{G}_{mm}$and the GraphSAGE output on the miRNA–disease association graph $\:{G}_{md}$, respectively. Similarly, let $\:{X}_{d}^{\left(1\right)}$and $\:{X}_{d}^{\left(2\right)}$denote two disease-view embedding matrices, which are obtained from the GraphSAGE output on the disease–disease similarity graph $\:{G}_{dd}$and the GraphSAGE output on the miRNA–disease association graph $\:{G}_{md}$, respectively.

We first concatenate the two embeddings and apply a linear transformation followed by a ReLU nonlinearity:

20 $$\:\begin{array}{cccc}&\:H=ReLU\left({W}_{1}\right[{X}_{1};{X}_{2}\left]\right)&\:&\:\end{array}$$

where $\:{W}_{1}$ is a learnable weight matrix and $\:\left[{X}_{1};{X}_{2}\right]$denotes concatenation.

Next, we compute an adaptive gating weight to modulate the contribution of each embedding source:

21 $$\:\begin{array}{cccc}&\:\alpha\:=\sigma\:\left({W}_{2}H\right)&\:&\:\end{array}$$

where $\:{W}_{2}$ is a learnable matrix and $\:\sigma\:(\cdot\:)$is the sigmoid function that maps values into $\:\left[0,1\right]$, enabling soft, context-dependent modulation.The fused representation is then obtained by:

22 $$\:\begin{array}{cccc}&\:Z=\alpha\:\odot\:{X}_{1}+(1-\alpha\:)\odot\:{X}_{2}&\:&\:\end{array}$$

where $\:\odot\:$ denotes element-wise multiplication (if $\:\alpha\:$ is computed as a node-wise scalar gate, it is broadcast across embedding dimensions).

We apply the above fusion operator separately to miRNA and disease embeddings. For miRNAs, given $\:{X}_{m}^{\left(1\right)}$(from the miRNA–miRNA similarity graph) and $\:{X}_{m}^{\left(2\right)}$(from the miRNA–disease association graph), we define the fused miRNA representation as:

23 $$\:{F}_{m}=Fuse({X}_{m}^{\left(1\right)},{X}_{m}^{\left(2\right)})$$

For diseases, given $\:{X}_{d}^{\left(1\right)}$(from the disease–disease similarity graph) and $\:{X}_{d}^{\left(2\right)}$(from the miRNA–disease association graph), we define the fused disease representation as:

24 $$\:{F}_{d}=Fuse({X}_{d}^{\left(1\right)},{X}_{d}^{\left(2\right)})$$

#### Global context fusion module

After completing the nonlinear adaptive fusion in Sect. (Nonlinear adaptive fusion of feature embeddings), we further introduce a global context fusion module to inject dataset-level global information and enhance representation robustness. Let $\:F\in\:{\mathbb{R}}^{N\times\:d}$denote the fused embedding matrix obtained in Sect. (Nonlinear adaptive fusion of feature embeddings) for a given entity type (i.e., $\:{F}_{m}$ for miRNAs and $\:{F}_{d}$ for diseases), where $\:N$ is the number of nodes of that entity type and $\:d$is the embedding dimension. We first apply global average pooling over all nodes to derive a compact global context vector:

25 $$\:\begin{array}{cccc}&\:g=\frac{1}{N}\sum\:_{i=1}^{N}{f}_{i}&\:&\:\end{array}$$

where $\:{\mathrm{f}}_{i}$denotes the embedding of the $\:i$-th node. The global context vector is then fed into a two-layer fully connected network, together with ReLU and sigmoid activations, to produce a global gating weight (a scalar):

26 $$\:\begin{array}{cccc}&\:{\alpha\:}_{\mathrm{global}}=\sigma\:\left({W}_{2}ReLU\left({W}_{1}g\right)\right)&\:&\:\end{array}$$

where $\:{W}_{1}$and $\:{W}_{2}$ are learnable parameters, and $\:\sigma\:(\cdot\:)$is the sigmoid function, ensuring $\:{\alpha\:}_{\mathrm{global}}\in\:\left[\mathrm{0,1}\right]$. Finally, we fuse the global context with each node embedding in a gated manner to obtain the global-enhanced node representations:

27 $$\:\begin{array}{cccc}&\:\stackrel{\sim}{F}={\alpha\:}_{\mathrm{global}}F+(1-{\alpha\:}_{\mathrm{global}})l{g}^{\top\:}&\:&\:\end{array}$$

where $\:l{g}^{\top\:}$broadcasts $\:\mathrm{g}$to all nodes. This module adaptively balances node-specific local information and dataset-level global context. In our model, we apply this operation separately to miRNA and disease embeddings, yielding $\:{\stackrel{\sim}{F}}_{m}$and $\:{\stackrel{\sim}{F}}_{d}$, which are then used for downstream prediction.

#### Morphology images feature extraction module

In this study, to extract features from the morphological images, we partitioned the images into three scales: 512 × 512, 256 × 256, and 16 × 16. During the cropping process, a sliding-window strategy with appropriately set overlapping regions was employed to ensure that no critical pathological area was overlooked. The determination of the overlapping regions was primarily based on the image dimensions, the distribution of key pathological features, and the specific requirements of subsequent tasks. Typically, a fixed-ratio approach was adopted: for instance, for 256 × 256 sub-images, a 50% overlap (i.e., a stride of 128 pixels) was implemented, whereas for 16 × 16 sub-images, a stride of 8 pixels was chosen. This approach not only preserves the integrity of the image edge information but also captures sufficient contextual details. Furthermore, if the pathological features are sparse or particularly significant, the overlap ratio can be increased (e.g., to 60% or higher) to ensure that every critical region is adequately covered.

After obtaining multi-scale patches, we employ a pretrained ResNet-18 backbone to extract feature embeddings from each scale, denoted as $\:{F}_{512}$, $\:{F}_{256}$, and $\:{F}_{16}$, respectively. To fuse information across scales, we adopt a cross-attention mechanism where the 512-scale feature acts as a global-context query and the finer-scale features (256 × 256 and 16 × 16) provide the key/value information to be selectively aggregated. The attention operation is defined as:

28 $$\:Attention\left(Q,K,V\right)=softmax\left(\frac{Q{K}^{T}}{\sqrt{d}}\right)V$$

where $\:d$ is the feature dimension. This design enables the model to adaptively weight fine-scale morphological cues conditioned on the global structural context captured at 512 × 512.

After computing the attention, we obtain a tensor of multi-scale features. We then average these features along the scale axis (256 × 256, 16 × 16, and 512 × 512) to get the final representation:

29 $$\:\stackrel{-}{F}=\:\frac{1}{N}\sum\:_{i=1}^{N}{Attention(Q,K,V)}_{i}$$

Finally, this fused feature vector is passed through a fully-connected (FC) layer, which projects it into a lower-dimensional space:

30 $$\:{F}_{img}=FC\left(\stackrel{-}{F}\right)$$

This process effectively integrates multi-scale information and leverages the multi-head attention mechanism to capture global contextual dependencies, thereby enhancing the overall expressive power of the extracted features.

#### Prediction module

In this module, we integrate the miRNA features $\:{\stackrel{\sim}{F}}_{m}$ and disease features $\:{\stackrel{\sim}{F}}_{d}$ obtained from the Global Context Fusion module with the image features $\:{\stackrel{\sim}{F}}_{img}$ extracted by the Morphology Images Feature Extraction module. The image features are incorporated as supplementary knowledge to further enhance model performance. The fused feature $\:{F}_{fused}$ is defined as:

31 $$\:{F}_{fused}=[{\stackrel{\sim}{F}}_{m},{\stackrel{\sim}{F}}_{d},{\stackrel{\sim}{F}}_{img}]$$

Subsequently, $\:{F}_{fused}$ is processed by a fully connected layer mapping function $\:\varnothing\:(\bullet\:)$ to generate the final prediction, which is formulated as:

32 $$\:{F}_{final}=\varnothing\:\left({F}_{fused}\right)$$

### Overall loss function

To effectively optimize EMGMDA, we adopt a training strategy that combines triplet contrastive learning with binary cross-entropy (BCE) supervision, enabling the model to learn discriminative embeddings while simultaneously improving prediction accuracy.

Triplet contrastive learning has been widely applied in deep representation learning and metric learning [49]. Its principle is to enforce relative distance constraints, such that embeddings of similar entities are pulled closer together, whereas embeddings of dissimilar ones are pushed further apart. In this study, each triplet consists of three elements: the anchor (a target miRNA), a positive sample (a disease experimentally confirmed to be linked to that miRNA), and a negative sample (a disease for which no link is reported). The learning objective requires the anchor to lie closer to its positive counterpart than to the negative one in the embedding space.

Since the miRNA–disease association graph is sparse, the sampling of positive and negative instances is critical. For each anchor miRNA $\:m$, we first define the positive set $\:\mathcal{P}\left(m\right)$as diseases that are connected to $\:m$by observed associations in the training graph only (excluding any validation/test edges to avoid information leakage). A positive disease $\:{d}^{+}$is uniformly sampled from $\:\mathcal{P}\left(m\right)$. The negative pool $\:\mathcal{N}\left(m\right)$is defined as all diseases that are not linked to $\:m$in the training graph. To reduce degree bias in sparse graphs, we adopt degree-balanced negative sampling, where negative diseases are sampled with probabilities inversely related to their node degrees. In addition, to avoid overly easy negatives, we optionally perform semi-hard negative mining within each mini-batch, selecting negative diseases that are relatively close to the anchor in the embedding space while still not being labeled as positives. If no semi-hard negatives are available in the batch, we fall back to degree-balanced random negatives. This strategy improves the effectiveness and stability of metric learning under sparse supervision.

Formally, the triplet loss is defined as:

33 $$\:\begin{array}{cccc}&\:{L}_{\mathrm{t}\mathrm{r}\mathrm{i}}=\mathrm{m}\mathrm{a}\mathrm{x}\left(0,\parallel\:{z}_{{m}_{i}}-{z}_{{d}^{+}}{\parallel\:}_{2}^{2}-\parallel\:{z}_{{m}_{i}}-{z}_{{d}^{-}}{\parallel\:}_{2}^{2}+\delta\:\right)&\:&\:\end{array}$$

where $\:{z}_{{m}_{i}}$is the embedding of the $\:i$-th miRNA, $\:{z}_{{d}^{+}}$is the embedding of a positively associated disease sampled from $\:\mathcal{P}\left({m}_{i}\right)$, and $\:{z}_{{d}^{-}}$corresponds to a negative disease sampled from $\:\mathcal{N}\left({m}_{i}\right)$. The parameter $\:\delta\:$denotes a margin that enforces a minimum distance gap between positive and negative pairs.

To further improve predictive performance, we integrate the triplet objective with a binary cross-entropy term that directly supervises the association prediction task. The overall loss function is expressed as:

34 $$\:{L}_{total}={L}_{BCE}+{L}_{tri}$$

The BCE component is defined as:

35 $$\:{L}_{BCE}=-\frac{1}{N}\sum\:_{i=1}^{N}\:\left({y}_{i}\mathrm{l}\mathrm{o}\mathrm{g}\left({\widehat{y}}_{i}\right)+(1-{y}_{i})\mathrm{l}\mathrm{o}\mathrm{g}(1-{\widehat{y}}_{i})\right)$$

Where $\:{y}_{i}$ is the ground truth label, and$\:{\widehat{y}}_{i}$is the predicted probability for the *i* -th sample.

By jointly optimizing BCE and triplet contrastive losses, EMGMDA not only discriminates associated miRNA–disease pairs from those without evidence of association, while shaping the latent space to preserve biologically relevant relationships. This integrated objective enhances both robustness and generalization, particularly under conditions of sparse and heterogeneous data.

### Experimental configuration

The development and training of the model were carried out using PyTorch (v2.2.2) and PyTorch Geometric (v2.3.0). Parameter optimization was performed using the Adam optimizer. To identify the most effective hyperparameter configuration for the EMGMDA model, we conducted a series of controlled experiments, exploring various settings. Specifically, we evaluated different configurations for the learning rate ({0.1, 0.01, 0.001, 0.0001}), dropout rate ({0.1, 0.3, 0.5, 0.7}), and applied L2 regularization (weight decay) with values such as 1e-3. After thorough evaluation, we found that the optimal configuration for the EMGMDA model was a learning rate of 0.0001, and a dropout rate of 0.3. Furthermore, to mitigate the risk of overfitting, we implemented early stopping based on validation performance. The training process was terminated if there was no improvement in the validation metric after 20 epochs, thereby ensuring the model did not overfit to the training data. Based on these experiments, we provide practical guidelines for parameter selection. We suggest tuning the learning rate and dropout first, as they are typically the most influential. Weight decay can be kept as a mild default regularizer, and early stopping can be used to further reduce the risk of overfitting. When applying EMGMDA to other datasets, smaller or sparser datasets may benefit from stronger regularization or reduced model capacity, whereas larger datasets often support higher-capacity configurations.

### Evaluation metrics

To evaluate the performance of the predictive model, this study employed several metrics, including accuracy (ACC), sensitivity (Sn), specificity (Sp), Matthews correlation coefficient (MCC), and F1-score. The formulas for calculating these metrics are as follows:

36 $$Precision=\frac{{TP}}{{TP+FP}}$$

37 $$\operatorname{Re} call=\frac{{TP}}{{TP+FN}}$$

38 $$ACC=\frac{{TP+TN}}{{TP+FN+TN{\mathrm{+}}FP}}$$

39 $$F1 - score=\frac{{2 \times TP}}{{2 \times TP+FN+FP}}$$

Predicting miRNA–disease associations is typically framed as a binary classification task. In this setting, true positives (TP) are positive cases that are correctly identified, whereas false positives (FP) are negatives that are incorrectly labeled as positive. Precision measures the proportion of TP among all predicted positives, while recall quantifies the fraction of actual positives that are recovered. These indicators offer a basic view of model behavior. To obtain a more balanced assessment, we also report the F1 score, i.e., the harmonic mean of precision and recall. Because accuracy can be unreliable under class imbalance, we further analyze precision–recall (PR) and receiver operating characteristic (ROC) curves. The ROC curve relates the true-positive rate (TPR) to the false-positive rate (FPR), and its area under the curve (AUC) summarizes discrimination ability. The PR curve depicts the trade-off between recall and precision, and the corresponding AUPRC serves as another key summary; higher AUC/AUPRC values indicate stronger predictive performance.

## Results

### Experimental protocol and performance evaluation

To comprehensively evaluate the robustness and generalization ability of EMGMDA, we conducted five-fold cross-validation (5-CV) on two widely used benchmarks, HMDD v2.0 and HMDD v3.2. Specifically, all experimentally validated miRNA–disease associations (positive pairs) were randomly partitioned into five approximately equal folds. For each run, four folds were used for training and the remaining fold was used for testing.In each fold, the miRNA–disease association graph was reconstructed using training positives only, i.e., test positive edges were removed from the association matrix/graph before model training. This setting ensures that the model does not observe any test associations during representation learning and parameter optimization.

Since HMDD provides confirmed associations but does not provide explicit negative labels, negative samples were generated by randomly sampling unknown miRNA–disease pairs that do not appear in HMDD. For each fold, we constructed the test set by combining the held-out positive pairs with an equal number of randomly sampled negative pairs (1:1), following common practice in miRNA–disease association prediction. The same sampling protocol was applied consistently across all compared methods to ensure a fair comparison.

For each fold, we computed AUC and AUPRC as threshold-independent metrics, and additionally reported ACC, F1-score, Recall, and Precision under a fixed decision threshold. Final results are reported as mean ± standard deviation across the five folds.

**Fig. 3:**
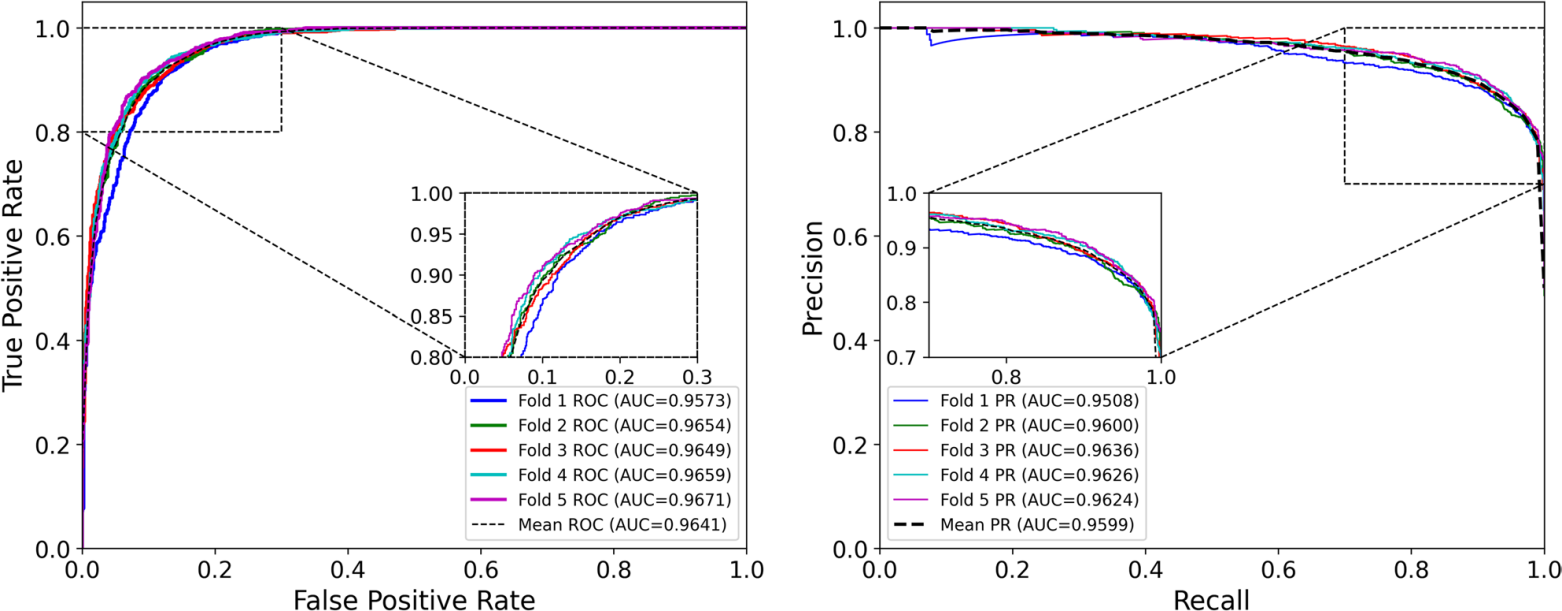
ROC and PR curves of EMGMDA model in five-fold cross-validation (HMDD v2.0 dataset)

**Fig. 4:**
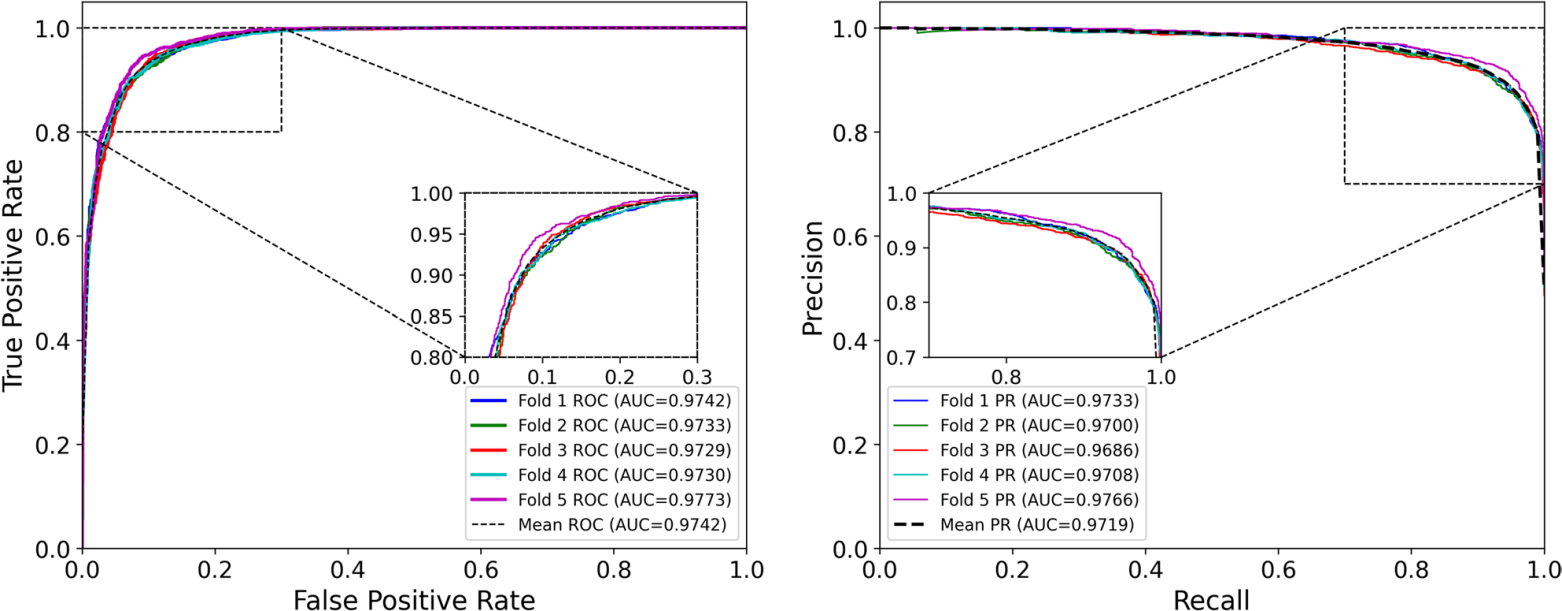
ROC and PR curves of EMGMDA model in five-fold cross-validation (HMDD v3.2 dataset)

As shown in Table 1, EMGMDA achieved strong predictive performance on HMDD v2.0, with an average AUC of 0.9641 ± 0.0035 and AUPRC of 0.9599 ± 0.0047, indicating stable performance across different data partitions. The ROC and PR curves of all five folds are shown in Fig. 3. On HMDD v3.2 (Table 2), EMGMDA further improved to an AUC of 0.9742 ± 0.0017 and an AUPRC of 0.9719 ± 0.0028, with consistently low variance across folds; the corresponding ROC/PR curves are presented in Fig. 4.

**Table 1:** Evaluation of EMGMDA with five-fold cross-validation on HMDD v2.0 dataset

Fold	AUC	AUPRC	ACC	F1-score	Recall	Precision
1	0.9573	0.9508	0.8923	0.8947	0.9061	0.8836
2	0.9654	0.9600	0.9001	0.8999	0.9242	0.8768
3	0.9649	0.9636	0.8964	0.9030	0.9424	0.8667
4	0.9659	0.9626	0.9042	0.9077	0.9437	0.8744
5	0.9671	0.9624	0.9061	0.9056	0.9030	0.9081
Average	0.9641 ± 0.0035	0.9599 ± 0.0047	0.8998 ± 0.0050	0.9022 ± 0.0046	0.9239 ± 0.0172	0.8819 ± 0.0142

**Table 2:** Evaluation of EMGMDA with five-fold cross-validation on HMDD v3.2

Fold	AUC	AUPRC	ACC	F1-score	Recall	Precision
1	0.9742	0.9733	0.9156	0.9198	0.9258	0.9163
2	0.9733	0.9700	0.9127	0.9166	0.9218	0.9093
3	0.9729	0.9686	0.9186	0.9216	0.9392	0.8980
4	0.9730	0.9708	0.9153	0.9219	0.9460	0.8963
5	0.9773	0.9766	0.9180	0.9230	0.9336	0.9223
Average	0.9742 ± 0.0017	0.9719 ± 0.0028	0.9180 ± 0.0052	0.9206 ± 0062	0.9336 ± 0.0125	0.9084.0101

### Comparison with state-of-the-art methods

To comprehensively evaluate the predictive capability of EMGMDA, we performed comparative experiments with multiple state-of-the-art methods. These competitors were selected because they are representative and influential approaches for miRNA–disease association prediction in recent years, cover diverse methodological paradigms (multimodal learning, Transformer-based architectures, spectral graph Transformers, and metapath-/heterogeneous hypergraph-based modeling), and have reported strong performance on widely used benchmarks such as HMDD v2.0 and/or HMDD v3.2. The first baseline, MINIMDA [48], improves prediction performance by leveraging higher-order neighborhood information of both miRNAs and diseases within a multimodal learning framework. The second competitor, MDformer [34], is a Transformer-based model that integrates features from multiple sources and has demonstrated strong performance on both HMDD v2.0 and HMDD v3.2. The third method, MHCLMDA [50], utilizes a multi-hypergraph contrastive learning scheme tailored for miRNA–disease association tasks. The fourth baseline, DARSFormer [51], combines spectral graph Transformers with a dynamic attention mechanism to enhance representation learning. Finally, MHMDA [52] adopts a multi-hop metapath strategy characterized by a “similarity–association–similarity” paradigm, incorporating hierarchical attention and a heterogeneous hypergraph network to capture long-range dependencies and latent associations, thereby achieving robust predictive accuracy. All methods were evaluated under the same five-fold splits and the same training/test construction protocol, including the same negative sampling strategy and evaluation procedure described in Sect. (Experimental protocol and performance evaluation). The complete list of parameter settings and implementation details for each competitor has been systematically summarized (see supplementary files for details).

**Fig. 5:**
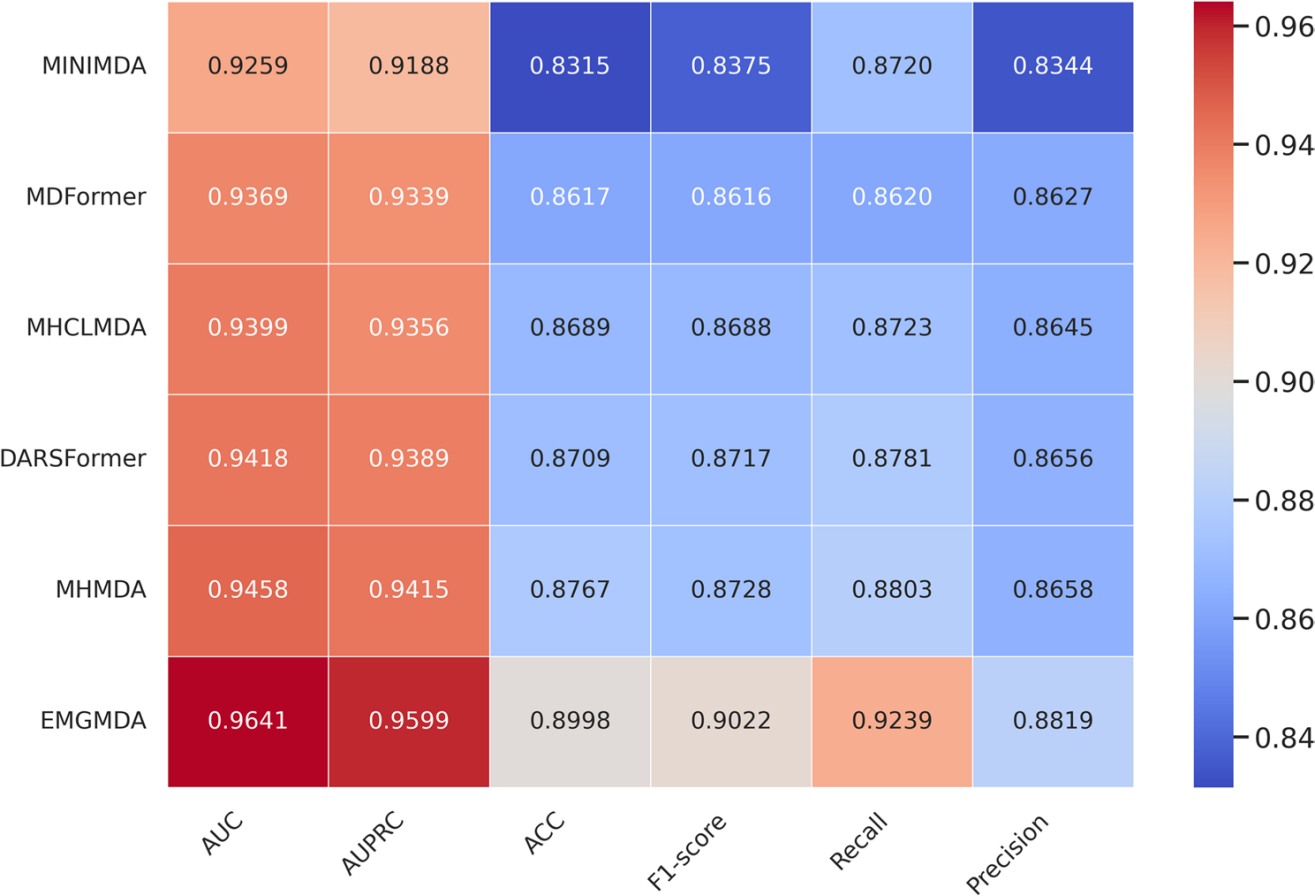
Cross-validation (five folds) results for the evaluated models on HMDD v2.0

**Fig. 6:**
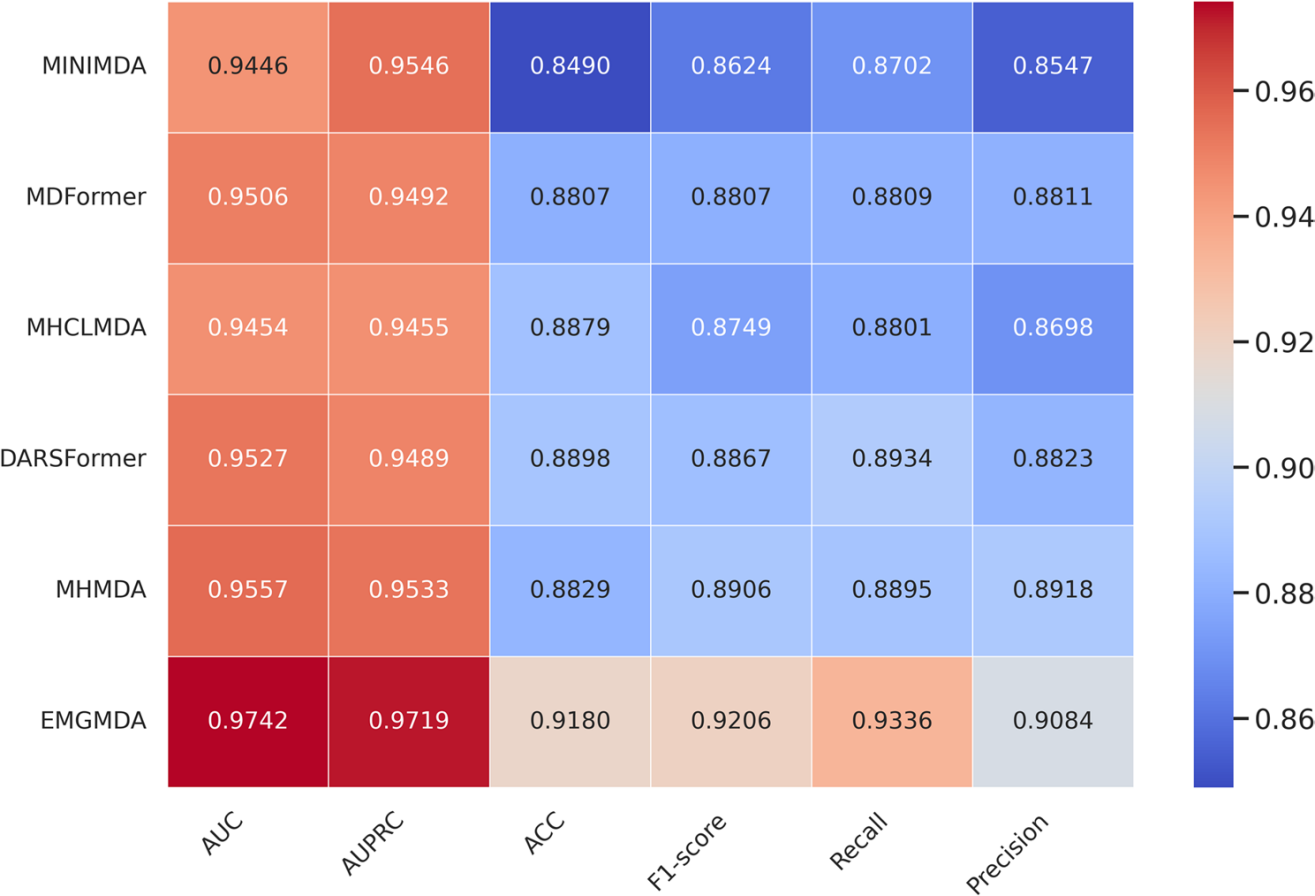
Cross-validation (five folds) results for the evaluated models on HMDD v3.2

On the HMDD v2.0 dataset (Fig. 5), EMGMDA achieves an AUC of 0.9641 and an AUPRC of 0.9599, outperforming MHMDA (0.9458/0.9415), DARSFormer (0.9418/0.9389), MHCLMDA (0.9399/0.9356), MDFormer (0.9369/0.9339), and MINIMDA (0.9259/0.9188). It also delivers the best accuracy (0.8998), F1-score (0.9022), recall (0.9239), and precision (0.8819), exceeding the strongest baseline by 1.6–4.4% points, which indicates superior discrimination and a well-balanced trade-off between sensitivity and specificity.On the HMDD v3.2 dataset (Fig. 6), EMGMDA further extends its lead, reaching an AUC of 0.9742 and an AUPRC of 0.9719, compared with MHMDA (0.9557/0.9533), DARSFormer (0.9527/0.9489), MDFormer (0.9506/0.9492), MHCLMDA (0.9454/0.9455), and MINIMDA (0.9446/0.9546). EMGMDA again ranks first in accuracy (0.9180), F1-score (0.9206), recall (0.9336), and precision (0.9084).Overall, these results demonstrate that EMGMDA consistently outperforms multiple state-of-the-art methods across both datasets, offering strong accuracy, robustness, and generalizability for miRNA–disease association prediction.

### Statistical significance analysis

To assess whether the improvements of EMGMDA over competing methods are statistically reliable, we conducted paired statistical significance tests using the fold-wise results obtained under the same five-fold cross-validation splits. For each dataset and metric (AUC and AUPRC), we computed the paired differences across folds, $\:{{\Delta\:}}_{k}={s}_{\mathrm{EMGMDA}}^{\left(k\right)}-{s}_{\mathrm{baseline}}^{\left(k\right)}$for $\:k=1,\dots\:,5$, and applied a two-sided paired t-test to evaluate the null hypothesis that the mean difference is zero. Because multiple baselines are compared simultaneously, we further controlled the family-wise error rate by applying the Holm–Bonferroni correction within each dataset–metric group, and report both raw and adjusted p-values.

For AUC, the significance results are summarized in Supplementary Material Table S1 and Table S2. For AUPRC, the significance results are summarized in Supplementary Material Table S3 and Table S4. The results show that EMGMDA achieves statistically significant improvements over all baselines: on HMDD v2.0, the Holm-adjusted *p*-values are < 0.01 for both AUC and AUPRC across all comparisons; on HMDD v3.2, EMGMDA is highly significant against all baselines on AUC (Holm-adjusted *p*-values < 0.001) and remains significant on AUPRC (Holm-adjusted p-values < 0.01, with comparisons against MDFormer and DARSFormer reaching Holm-adjusted *p*-values < 0.001). Overall, these fold-wise paired tests, together with multiple-comparison control, provide statistical evidence that the observed gains are robust and not attributable to a favorable data split.

### Cold-start evaluation experiment

To further evaluate the robustness and real-world applicability of EMGMDA, we conducted cold-start experiments under two challenging yet realistic scenarios: miRNA cold-start and disease cold-start.he choice of datasets for the cold-start experiments is based on the distinct characteristics of each dataset. HMDD v2.0 (495 miRNAs, 383 diseases) was used for miRNA cold-start to evaluate EMGMDA’s performance on a moderate-sized, balanced dataset with a limited number of miRNAs. On the other hand, HMDD v3.2 (853 miRNAs, 591 diseases) was chosen for disease cold-start to assess the model’s robustness with a larger and more complex dataset, more representative of real-world scenarios where unseen diseases are common. This approach allows for a comprehensive evaluation of EMGMDA’s generalizability across different scales.In the miRNA cold-start setting, all miRNAs were first randomly partitioned into five non-overlapping subsets. In each fold, one subset of miRNAs was selected as the test set, and all miRNA–disease associations involving these miRNAs were completely removed from the training data and used exclusively for testing. Consequently, the miRNAs appearing in the test set were entirely unseen during training.Similarly, in the disease cold-start setting, diseases were randomly divided into five disjoint subsets, and in each fold, one subset of diseases was held out. All associations involving the held-out diseases were excluded from the training set and reserved for testing, ensuring that the tested diseases did not appear in the training phase. For both cold-start scenarios, we adopted node-level five-fold cross-validation, where each subset was used once for testing and the remainder for training. Negative samples were generated using the same random sampling strategy as in Sect. (Experimental protocol and performance evaluation), applied after the cold-start split to prevent information leakage. All results were averaged over five folds with identical random seeds to reduce randomness.

Under the disease cold-start setting on HMDD v2.0 (Table S5), EMGMDA achieves an AUC of 0.9154 and an AUPRC of 0.9087, outperforming the strongest baseline MHMDA by approximately 2.2% in AUC and 1.9% in AUPRC. EMGMDA also attains higher ACC, F1-score, Recall, and Precision, indicating more reliable generalization to unseen diseases. Similarly, under the miRNA cold-start setting on HMDD v3.2 (Table S6), EMGMDA attains the highest AUC (0.9289) and AUPRC (0.9234), surpassing the best competing method by around 2.0%–2.5%, with consistent improvements across all other metrics. Overall, these results demonstrate that EMGMDA is more robust to data sparsity and cold-start conditions than existing methods. This advantage stems from its effective integration of multimodal representations and global relational modeling, making it well suited for real-world scenarios where newly identified miRNAs or diseases lack sufficient known associations.

### Hyperparameter experiment

In this study, we explored the impact of various hyperparameters on the performance of EMGMDA on the HMDDv2.0 and HMDDv3.2 datasets. To ensure robustness, we conducted five-fold cross-validation and tuned key hyperparameters—$\:\mathrm{K}$ in KNN, the number of GraphSAGE layers, and embedding sizes (32, 64, 128, 256). With the optimal settings, the model achieved the best performance on both datasets. Each experiment altered one hyperparameter at a time, keeping others fixed to precisely assess its effect on model performance.

**Fig. 7:**
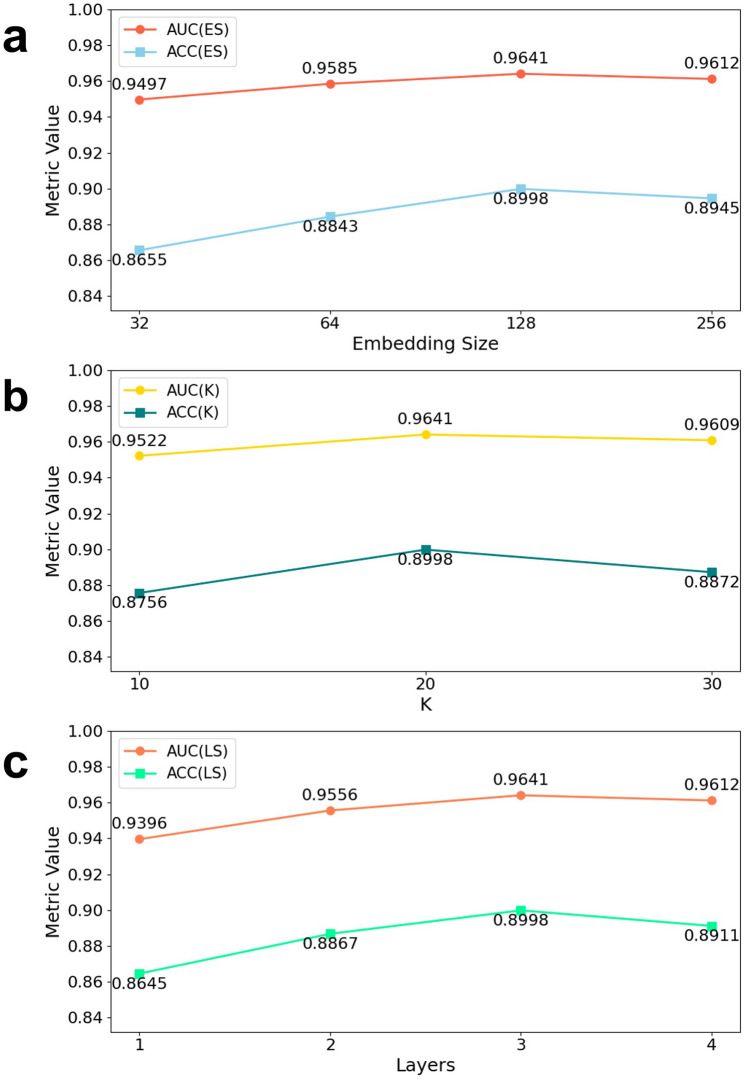
Performance of EMGMDA on the HMDDv2.0 dataset across different hyperparameter configurations. **a** Effect of embedding size on AUC and ACC; **b** Effect of K in KNN on AUC and ACC; **c** Effect of the number of GraphSAGE layers on AUC and ACC

**Fig. 8:**
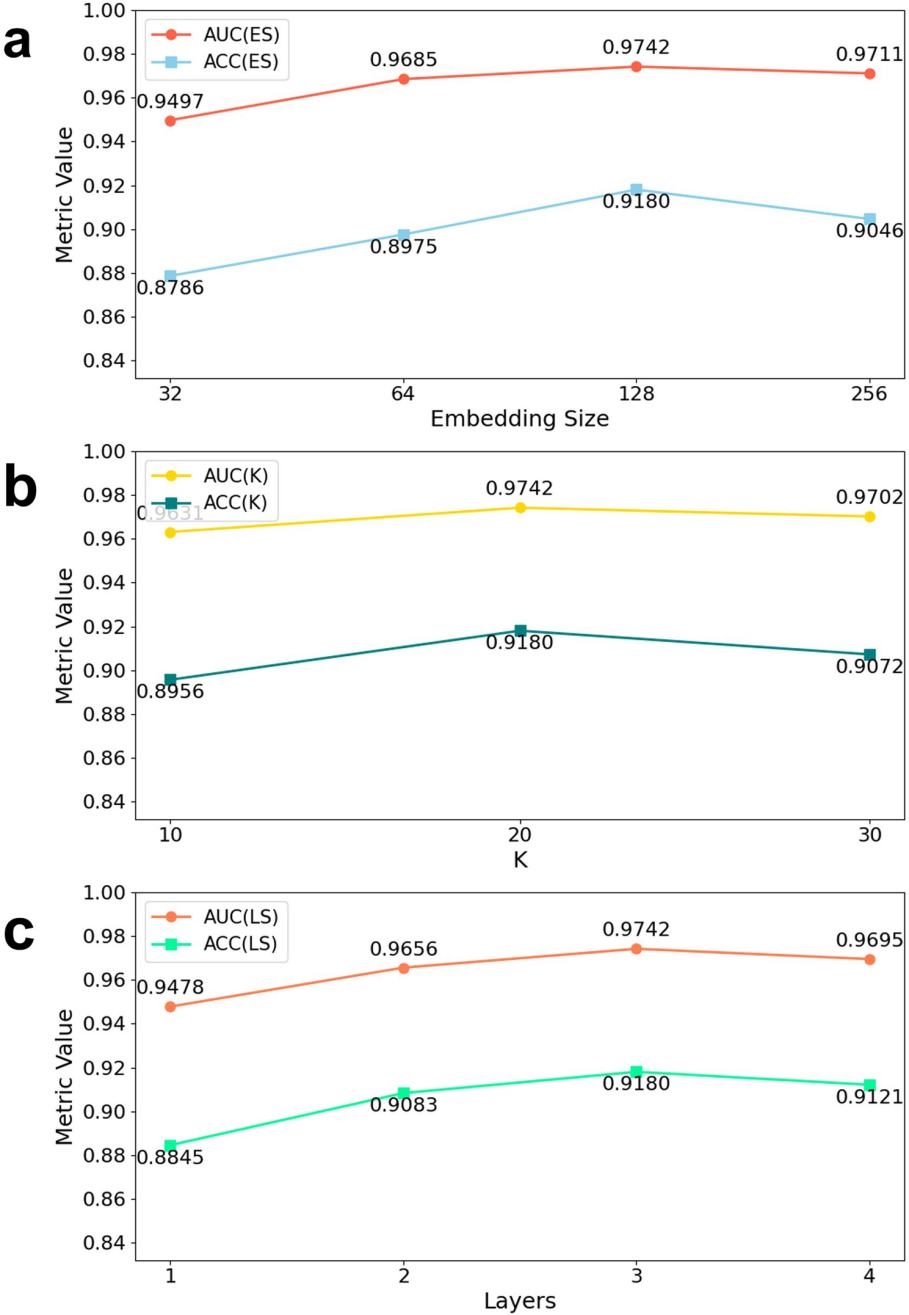
Performance of EMGMDA on the HMDDv3.2 dataset across different hyperparameter configurations. **a** Effect of embedding size on AUC and ACC; **b** Effect of K in KNN on AUC and ACC; **c** Effect of the number of GraphSAGE layers on AUC and ACC

The results indicate that increasing the embedding size improves performance on both datasets. Figures 7a and 8a show that increasing the embedding size from 32 to 128 steadily improves AUC(ES) and ACC(ES), rising from 0.9497 to 0.9641 and from 0.8655 to 0.8998 on v2.0, and from 0.9497 to 0.9742 and from 0.8786 to 0.9180 on v3.2, before both metrics dip slightly at 256 dimensions. Figures 7b and 8b demonstrate that a KNN neighborhood size of 20 maximizes AUC(K)/ACC(K) (0.9641/0.8998 on v2.0; 0.9742/0.9180 on v3.2), with only marginal declines at larger K. Figures 7c and 8c reveal that a three-layer GraphSAGE backbone yields the best discrimination (AUC = 0.9641/0.9742; ACC = 0.8998/0.9180), whereas adding a fourth layer produces slight performance drops. Together, these sweeps identify a single hyperparameter configuration (embedding size 128, K = 20, three GraphSAGE layers) that consistently maximizes EMGMDA’s discriminative power and accuracy across both HMDD benchmarks.

### Evaluation of model robustness for miRNA–disease association prediction across varying sample ratios

In practical miRNA–disease association prediction, the number of experimentally confirmed associations (positives) is far smaller than the number of unobserved pairs. Notably, the “unknown” associations in HMDD are essentially unlabeled rather than true negatives, meaning that some sampled negative pairs may actually correspond to undiscovered positives (i.e., false negatives). This positive–unlabeled characteristic, together with extreme class imbalance, can bias evaluation if only accuracy is considered. Therefore, besides AUC, we emphasize AUPRC, which is more informative under imbalance and better reflects the model’s ability to retrieve true associations from a large pool of unknown pairs. To simulate realistic imbalance and to assess robustness against potential sampling bias, we evaluate EMGMDA under multiple positive-to-negative ratios (1:1, 1:2, 1:5, 1:10). As expected, increasing the proportion of negatives leads to performance degradation, with AUPRC being more sensitive than AUC. Nevertheless, EMGMDA maintains stable AUC and competitive AUPRC across ratios, indicating robustness to imbalance.

**Fig. 9:**
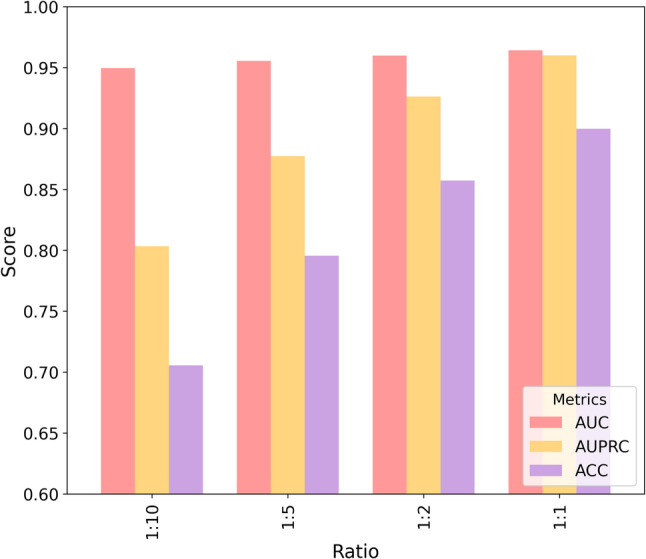
Performance of EMGMDA on the HMDD v2.0 dataset under different positive-to-negative sample ratios

**Fig. 10:**
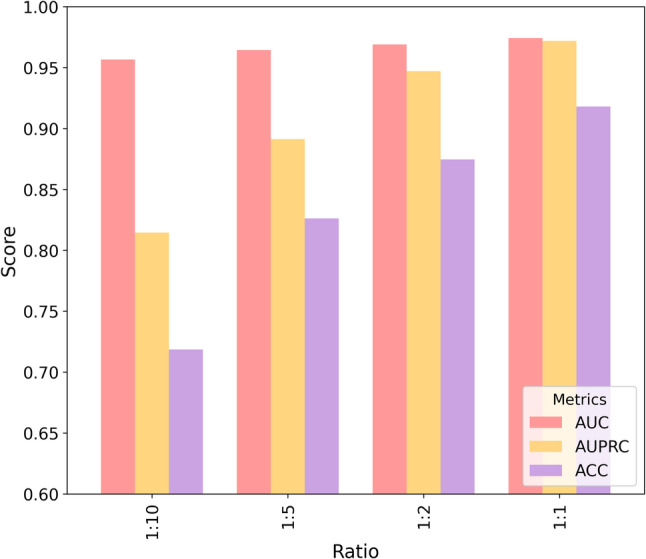
Performance of EMGMDA on the HMDD v3.2 dataset under different positive-to-negative sample ratios

On the HMDD v2.0 dataset (Fig. 9), as the ratio decreases from 1:1 to 1:10, EMGMDA’s AUC drops only slightly from 0.9641 to 0.9495, indicating stable overall discriminative ability, whereas AUPRC declines more noticeably from 0.9599 to 0.8033, suggesting higher sensitivity to class imbalance, and ACC shows the largest decrease from 0.8998 to 0.7055 due to the dominance of negative samples. On the HMDD v3.2 dataset (Fig. 10), EMGMDA consistently performs better, achieving its best results under the 1:1 ratio with an AUC of 0.9742, an AUPRC of 0.9719, and an ACC of 0.9180; even under the 1:10 ratio, it maintains strong predictive performance with an AUC of 0.9566, an AUPRC of 0.8144, and an ACC of 0.7186. Overall, these results indicate that EMGMDA is relatively robust to class imbalance and benefits from larger datasets, with AUC remaining the most stable metric, AUPRC being more sensitive to imbalance, and ACC exhibiting the greatest variability, supporting its robustness and practical utility for miRNA–disease association prediction.

### Ablation experiment

In this study, we conducted ablation experiments to quantify the contribution of key components in EMGMDA. We performed five-fold cross-validation on HMDD v2.0 and HMDD v3.2, and evaluated the methods using AUC, AUPR, Precision, Recall, F1-score, and Accuracy. We compared the full EMGMDA with five variants: EMGMDA-sim (using only the miRNA–disease similarity network), EMGMDA-ass (using only the miRNA–disease association network), EMGMDA-nim (removing histopathological image features), EMGMDA-nnf (replacing the nonlinear adaptive fusion module with direct concatenation), and EMGMDA-nts (removing the triplet contrastive loss and optimizing only with classification loss).

**Fig. 11:**
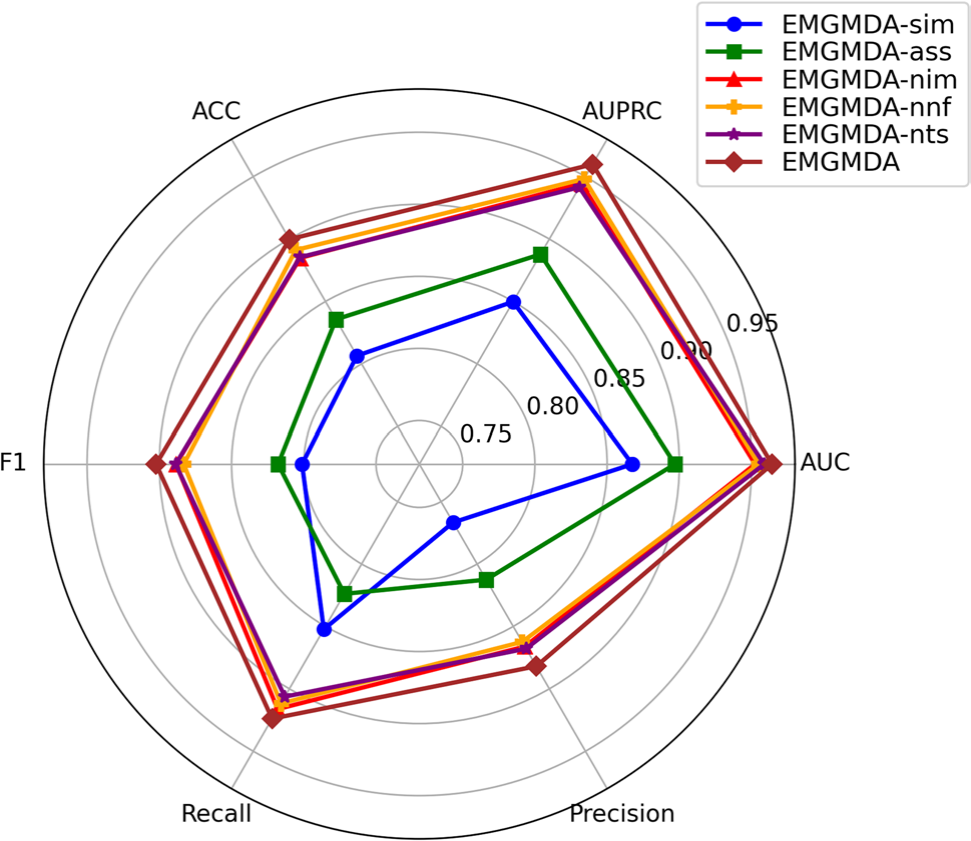
Comparative results of different models in ablative experiments on the HMDD v2.0 dataset

**Fig. 12:**
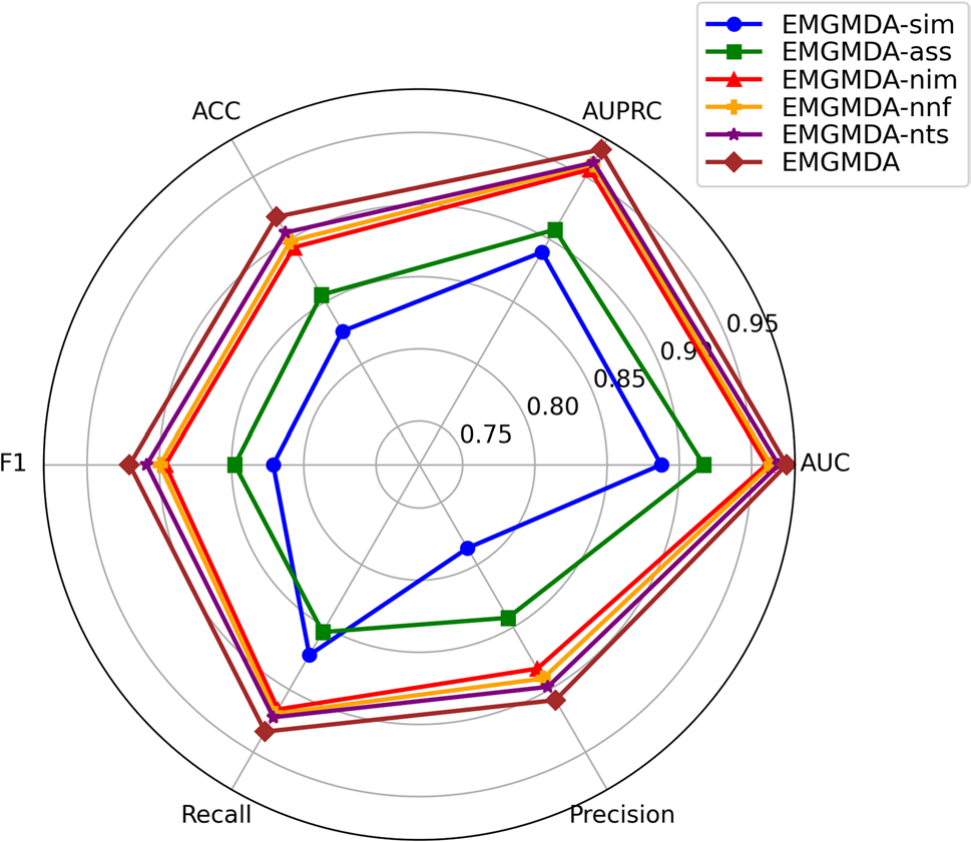
Comparative results of different models in ablative experiments on the HMDD v3.2 dataset

The full quantitative results are shown in Fig. 11 (HMDD v2.0) and Fig. 12 (HMDD v3.2). Overall, EMGMDA achieves the best performance on both datasets (HMDD v2.0: AUC/AUPR = 0.9641/0.9599; HMDD v3.2: 0.9742/0.9719). By contrast, the single-modality variants (EMGMDA-sim and EMGMDA-ass) perform markedly worse, indicating that a single feature source is insufficient.Ablation studies further verify the contribution of each component. Removing image-derived morphology features (EMGMDA-nim) consistently degrades performance (HMDD v2.0: 0.9524/0.9445; HMDD v3.2: 0.9604/0.9560), suggesting that histopathological cues provide complementary phenotype information. Replacing nonlinear fusion with simple concatenation (EMGMDA-nnf) also reduces AUC/AUPR (HMDD v2.0: 0.9549/0.9482; HMDD v3.2: 0.9634/0.9601), highlighting the benefit of nonlinear adaptive fusion for modeling cross-modal interactions. Similarly, removing triplet contrastive learning (EMGMDA-nts) lowers performance (HMDD v2.0: 0.9587/0.9415; HMDD v3.2: 0.9687/0.9615), supporting its role in improving discrimination under sparse supervision.

Overall, these results confirm that multimodal inputs, nonlinear fusion, and triplet contrastive learning each contribute substantially to EMGMDA’s effectiveness.

### Case study

To evaluate the practical utility of EMGMDA, we performed case studies on four common cancers: lung, esophageal, breast, and colorectal. For each cancer type, training data were constructed by combining validated miRNA–disease associations (positive samples) with an equal number of unconfirmed pairs (negative samples). After training, the model was used to prioritize candidate miRNA–disease interactions for the target cancer, and the top 50 candidates with the highest prediction scores were shortlisted.Validation was conducted using dbDEMC [53] and miR2Disease [54], where evidence recorded in dbDEMC was considered sufficient for confirmation. To prevent trivial information leakage during ranking, we adopted a consistent leave-one-out protocol: candidate miRNA–disease pairs evaluated in the case study were excluded from the training edges when generating the ranked list.

In the first case study, esophageal cancer, a malignancy developing in the esophagus [55–57], was examined. EMGMDA predicted 50 candidate miRNAs, all of which were verified using both the dbDEMC [53] and miR2Disease [54] databases (Supporting Material Table S7). The second case addressed breast cancer, a prevalent global disease affecting primarily women but also occurring in men [58–60]. Again, all 50 predicted miRNAs were corroborated by dbDEMC (Supporting Material Table S8). The third case focused on lung cancer, a major cause of cancer mortality worldwide [61], typically categorized into NSCLC and SCLC [62]. All 50 miRNA predictions were confirmed via dbDEMC (Supporting Material Table S9). The final case investigated colorectal cancer (CRC), a common gastrointestinal malignancy [63]. Among the top 50 predictions, 49 were validated through dbDEMC (Supporting Material Table S10). Although hsa-miR-449a was not listed in the database, its role in CRC had been previously established by Ishikawa et al. [64], lending additional credibility to the prediction. These case studies across four major cancers illustrate the consistent accuracy and practical utility of EMGMDA in identifying miRNA–disease associations.

To isolate the contribution of histopathological image features in the case study, we additionally report results under the same split for three settings: (i) EMGMDA (full), (ii) EMGMDA-nim, which removes image features, and (iii) an image-control setting where image features are randomly permuted across diseases while keeping the graph structure unchanged. The top-50 validation statistics under these three settings are summarized in Supporting Material Table S11, which quantifies the marginal benefit contributed by pathology features. Overall, these case studies across four cancers demonstrate the practical utility of EMGMDA in prioritizing plausible miRNA–disease associations. Importantly, the ablation and control comparisons further support that incorporating histopathological image features provides complementary predictive evidence beyond network information alone, while the permuted-image control indicates that the gain is attributable to meaningful disease-aligned pathology signals rather than incidental feature augmentation.

## Discussion

MGMDA advances miRNA–disease association prediction through four key innovations: residual GraphSAGE for robust embedding extraction, nonlinear adaptive fusion to capture high-order interactions, multi-scale histopathological image integration via ResNet-18, and triplet contrastive learning for enhanced discrimination. These innovations collectively enable the model to achieve AUCs of 0.9641 and 0.9742 on HMDD v2.0 and v3.2, respectively. Notably, the dynamic fusion module avoids redundancy inherent in simple concatenation, while residual connections preserve critical signals in sparse networks. Histopathological features contribute complementary morphological cues that substantially enhance performance, providing an added layer of biological context that purely network-based models lack. The case studies across four cancer types (esophageal, breast, lung, and colorectal) demonstrated strong validation for top predictions in esophageal, breast, and lung cancers, with reliable performance in colorectal cancer, further supporting the clinical utility of EMGMDA.

Despite the strong performance of EMGMDA, several limitations should be noted. First, the model is inherently constrained by the completeness and quality of its input similarity and association networks. Mechanisms not encoded in these networks, such as gene expression dynamics, epigenetic regulation, microenvironmental influences, or environmental exposures, may therefore be underrepresented. In this work, the histopathology branch is not meant to claim discovery of entirely novel pathological entities, but to provide complementary tissue-level phenotypic cues extracted from whole-slide images that supplement network-based molecular associations. Future extensions could incorporate additional omics modalities (e.g., transcriptomics, proteomics, methylation) to broaden mechanistic coverage, though this will require careful fusion strategies to handle heterogeneous scales and noise.Second, our histopathological analysis is limited to five major TCGA cancers, which may restrict generalizability to rare cancers, non-oncological diseases, or real-world multi-center cohorts. Addressing data scarcity and domain shift (e.g., staining and scanner variability) will be essential, potentially via transfer learning, domain adaptation, or federated learning. Third, miRNA–disease association prediction inherently suffers from severe class imbalance, and “unknown” miRNA–disease pairs in curated databases are unlabeled rather than confirmed negatives. As a result, random negative sampling may introduce false negatives, which can bias model training and lead to overly optimistic performance estimates. This limitation may reduce the robustness and real-world applicability of prediction models when deployed on unseen or incomplete biological data. Accordingly, incorporating more principled strategies, such as positive–unlabeled (PU) learning, hard-negative mining, or biologically guided reliable-negative construction, represents a promising direction for mitigating this issue and further improving real-world applicability. Finally, interpretability remains a barrier to clinical adoption. Combining post-hoc explanations (e.g., attention visualization and gradient-based attribution for images) with graph explainers or interpretable GNN variants could help identify the morphological regions and network neighborhoods most responsible for predictions. From a scalability standpoint, more efficient graph construction and sampling strategies may be needed as datasets expand.In conclusion, EMGMDA advances miRNA–disease association prediction through multi-modal fusion and contrastive learning, where histopathology contributes complementary phenotype information within the limits of available data sources. Further improvements in generalization, and interpretability will enhance its clinical and translational potential.

## Conclusions

EMGMDA addresses critical challenges in miRNA–disease association prediction by integrating residual GraphSAGE, nonlinear adaptive fusion, multi-scale histopathological features, and triplet contrastive learning, achieving state-of-the-art performance on HMDD v2.0 and HMDD v3.2 with AUCs of 0.9641 and 0.9742, respectively. Case studies across four major cancers further demonstrate its practical value in prioritizing candidate miRNAs for biomarker discovery. Despite these encouraging results, several limitations remain, including dependence on the completeness of similarity/association networks, the restricted coverage of TCGA cancer types, and limited interpretability for clinical deployment. Looking forward, future work in miRNA–disease association prediction will likely focus on integrating richer evidence such as multi-omics data and literature-derived knowledge to improve mechanistic coverage, enhancing generalization to rare diseases and real-world cohorts through transfer learning and domain adaptation, better handling positive–unlabeled and class-imbalance settings via PU learning, reliable-negative construction, and uncertainty-aware modeling, systematically evaluating and improving few-shot performance for newly discovered miRNAs or diseases, and developing scalable as well as explainable AI techniques to support biological interpretation and wet-lab validation. By providing a robust, accurate, and clinically relevant framework, EMGMDA establishes a foundation for advancing miRNA therapeutics and personalized medicine.

## Supplementary Information

Supplementary Material 1.

## Data Availability

The datasets and source code supporting this research are publicly accessible at [https://github.com/SJNNNN/EMGMDA](https:/github.com/SJNNNN/EMGMDA) . The miRNA–disease association data were obtained from the Human MicroRNA Disease Database (HMDD) (https://www.cuilab.cn/hmdd/), including benchmark datasets derived from HMDD v2.0 and v3.2. Histopathological whole-slide images were obtained from The Cancer Genome Atlas (TCGA) via the Genomic Data Commons (GDC) portal: https://portal.gdc.cancer.gov/.

## Abbreviations

AUC: Area Under the Curve
AUPRC: Area Under the Precision-Recall Curve
BCE: Binary Cross-entropy
GIP: Gaussian Interaction Profile
GNNs: Graph Neural Networks
HMDD: Human MicroRNA-Disease Database
KNN: K-Nearest Neighbor
MeSH: Medical Subject Headings
miRNA: microRNA
PR: Precision-Recall
ROC: Receiver Operating Characteristic
TCGA: The Cancer Genome Atlas

## References

[1] Ambros V. MicroRNAs: Tiny regulators with great potential. Cell. 2001;107:823–6. 11779458 10.1016/s0092-8674(01)00616-x

[2] Ambros V. MicroRNA pathways in flies and worms: Growth, death, fat, stress, and timing. Cell. 2003;113:673–6. 12809598 10.1016/s0092-8674(03)00428-8

[3] Li T, Morgan MJ, Choksi S, Zhang Y, Kim YS, Liu Z. MicroRNAs modulate the noncanonical transcription factor NF-κB pathway by regulating expression of the kinase IKKα during macrophage differentiation. Nat Immunol. 2010;11:799–805. 20711193 10.1038/ni.1918 PMC2926307

[4] Alshalalfa M, Alhajj R. Using context-specific effect of miRNAs to identify functional associations between miRNAs and gene signatures. BMC Bioinformatics. 2013;14:S1. 24267745 10.1186/1471-2105-14-S12-S1 PMC3848857

[5] Lou K, Chen N, Li Z, Zhang B, Wang X, Chen Y, Xu H, Wang D, Wang H. MicroRNA-142-5p overexpression inhibits cell growth and induces apoptosis by regulating FOXO in hepatocellular carcinoma cells. Oncol Res. 2017;25:65. 28081734 10.3727/096504016X14719078133366 PMC7840786

[6] Mattie MD, Benz CC, Bowers J, Sensinger K, Wong L, Scott GK, Fedele V, Ginzinger D, Getts R, Haqq C. Optimized high-throughput microRNA expression profiling provides novel biomarker assessment of clinical prostate and breast cancer biopsies. Mol Cancer. 2006;5:24. 16784538 10.1186/1476-4598-5-24 PMC1563474

[7] Schmittgen TD, Lee EJ, Jiang J, Sarkar A, Yang L, Elton TS, Chen C. Real-time PCR quantification of precursor and mature microRNA. Methods. 2008;44:31–8. 18158130 10.1016/j.ymeth.2007.09.006 PMC2663046

[8] Baskerville S, Bartel DP. Microarray profiling of microRNAs reveals frequent coexpression with neighboring miRNAs and host genes. RNA. 2005;11:241–7. 15701730 10.1261/rna.7240905 PMC1370713

[9] Chen X, Xie D, Zhao Q, You ZH. MicroRNAs and complex diseases: from experimental results to computational models. Brief Bioinform. 2019;20(2):515–39. 29045685 10.1093/bib/bbx130

[10] Huang L, Zhang L, Chen X. Updated review of advances in microRNAs and complex diseases: taxonomy, trends and challenges of computational models. Brief Bioinform. 2022;23(5):bbac358. 36056743 10.1093/bib/bbac358

[11] Huang L, Zhang L, Chen X. Updated review of advances in microRNAs and complex diseases: towards systematic evaluation of computational models. Brief Bioinform. 2022;23(6):bbac407. 36151749 10.1093/bib/bbac407

[12] Huang L, Zhang L, Chen X. Updated review of advances in microRNAs and complex diseases: experimental results, databases, webservers and data fusion. Brief Bioinform. 2022;23(6):bbac397. 36094095 10.1093/bib/bbac397

[13] Jiang Q, Hao Y, Wang G, Juan L, Zhang T, Teng M, Liu Y, Wang Y. Prioritization of disease microRNAs through a human phenome-microRNAome network. BMC Syst Biol. 2010;4:S2. 20522252 10.1186/1752-0509-4-S1-S2 PMC2880408

[14] Chen X, Yan CC, Zhang X, You ZH, Deng L, Liu Y, Zhang Y, Dai Q. WBSMDA: Within and between score for miRNA-disease association prediction. Sci Rep. 2016;6:21106. 26880032 10.1038/srep21106 PMC4754743

[15] Xu J, Li CX, Lv JY, Li YS, Xiao Y, Shao TT, Huo X, Li X, Zou Y, Han QL, Li X, Wang LH, Ren H. Prioritizing candidate disease miRNAs by topological features in the miRNA target-dysregulated network: Case study of prostate cancer. Mol Cancer Ther. 2011;10:1857–66. 21768329 10.1158/1535-7163.MCT-11-0055

[16] Chen X, Wang CC, Yin J, You ZH. Novel human miRNA-disease association inference based on random forest. Mol Ther Nucleic Acids. 2018;13:568–79. 30439645 10.1016/j.omtn.2018.10.005 PMC6234518

[17] Chen X, Yan CC, Zhang X, Li Z, Deng L, Zhang Y, Dai Q. RBMMMDA: Predicting multiple types of disease-microRNA associations. Sci Rep. 2015;5:13877. 26347258 10.1038/srep13877 PMC4561957

[18] Wang L, You ZH, Chen X, Li YM, Dong YN, Li LP, Zheng K. LMTRDA: Using logistic model tree to predict miRNA-disease associations by fusing multi-source information of sequences and similarities. PLoS Comput Biol. 2019;15:e1006865. 30917115 10.1371/journal.pcbi.1006865 PMC6464243

[19] Chen X, Li TH, Zhao Y, Wang CC, Zhu CC. Deep-belief network for predicting potential miRNA-disease associations. Brief Bioinform. 2021;22(3):bbaa186. 34020550 10.1093/bib/bbaa186

[20] Ji C, Gao Z, Ma X, Wu Q, Ni J, Zheng C. AEMDA: Inferring miRNA-disease associations based on deep autoencoder. Bioinformatics. 2021;37:66–72. 32726399 10.1093/bioinformatics/btaa670

[21] Liu W, Lin H, Huang L, Peng L, Tang T, Zhao Q, Yang L. Identification of miRNA-disease associations via deep forest ensemble learning based on autoencoder. Brief Bioinform. 2022;23:bbac104. 35325038 10.1093/bib/bbac104

[22] Xu J, Cai L, Liao B, Zhu W, Wang P, Meng Y, Lang J, Tian G, Yang J. Identifying potential miRNAs-disease associations with probability matrix factorization. Front Genet. 2019;10:1234. 31921290 10.3389/fgene.2019.01234 PMC6918542

[23] Cui L, Lu Y, Sun J, Fu Q, Xu X, Wu H, Chen J. RFLMDA: A novel reinforcement learning-based computational model for human microRNA-disease association prediction. Biomolecules. 2021;11:1835. 34944479 10.3390/biom11121835 PMC8699433

[24] Réau M, Renaud N, Xue LC, Bonvin AMJJ. DeepRank-GNN: A graph neural network framework to learn patterns in protein–protein interfaces. Bioinformatics. 2023;39:btac759. 36420989 10.1093/bioinformatics/btac759 PMC9805592

[25] Shen ZA, Luo T, Zhou YK, Yu H, Du PF. NPI-GNN: Predicting ncRNA–protein interactions with deep graph neural networks. Brief Bioinform. 2021;22:bbab051. 33822882 10.1093/bib/bbab051

[26] Gao Z, Jiang C, Zhang J, Jiang X, Li L, Zhao P, Yang H, Huang Y, Li J. Hierarchical graph learning for protein–protein interaction. Nat Commun. 2023;14:1093. 36841846 10.1038/s41467-023-36736-1 PMC9968329

[27] Li M, Liu Y, Wong BCL, Gan VJL, Cheng JCP. Automated structural design optimization of steel reinforcement using graph neural network and exploratory genetic algorithms. Autom Constr. 2023;146:104677.

[28] Chu Y, Wang X, Dai Q, Wang Y, Wang Q, Peng S, Wei X, Qiu J, Salahub DR, Xiong Y, Wei DQ. MDA-GCNFTG: identifying miRNA-disease associations based on graph convolutional networks via graph sampling through the feature and topology graph. Brief Bioinform. 2021;22(6):bbab165. 34009265 10.1093/bib/bbab165

[29] Tang X, Luo J, Shen C, Lai Z. Multi-view multichannel attention graph convolutional network for miRNA-disease association prediction. Brief Bioinform. 2021;22:bbab174. 33963829 10.1093/bib/bbab174

[30] Li Z, Li J, Nie R, You ZH, Bao W. A graph auto-encoder model for miRNA–disease associations prediction. Brief Bioinform. 2021;22:bbaa240. 34293850 10.1093/bib/bbaa240

[31] Ding Y, Tian LP, Lei X, Liao B, Wu FX. Variational graph auto-encoders for miRNA–disease association prediction. Methods. 2021;192:25–34. 32798654 10.1016/j.ymeth.2020.08.004

[32] Wang W, Chen H. Predicting miRNA-disease associations based on lncRNA–miRNA interactions and graph convolution networks. Brief Bioinform. 2023;24:bbac495. 36526276 10.1093/bib/bbac495

[33] Zhang H, Fang J, Sun Y, Xie G, Lin Z, Gu G. Predicting miRNA–disease associations via node-level attention graph auto-encoder. IEEE/ACM Trans Comput Biol Bioinform. 2023;20:1308–18. 35503834 10.1109/TCBB.2022.3170843

[34] Dong B, Sun W, Xu D, Wang G, Zhang T, MDformer:. A transformer-based method for predicting miRNA–disease associations using multi-source feature fusion and maximal meta-path instances encoding. Comput Biol Med. 2023;167:107585. 37890424 10.1016/j.compbiomed.2023.107585

[35] Li Z, Wan L, Wang L, Wang W, Nie R. HHOMR: A hybrid high-order moment residual model for miRNA-disease association prediction. Brief Bioinform. 2024;25:bbae412. 39175132 10.1093/bib/bbae412 PMC11341279

[36] Zhao BW, He YZ, Su XR, Yang Y, Li GD, Huang YA, Hu PW, You ZH, Hu L. Motif-aware miRNA–disease association prediction via hierarchical attention network. IEEE J Biomed Health Inf. 2024;28:4281–94. 10.1109/JBHI.2024.3383591 38557614

[37] Tian Z, Han C, Xu L, Teng Z, Song W. MGCNSS: miRNA–disease association prediction with multi-layer graph convolution and distance-based negative sample selection strategy. Brief Bioinform. 2024;25(3):bbae168. 38622356 10.1093/bib/bbae168 PMC11018511

[38] Fridman WH, Pagès F, Sautès-Fridman C, Galon J. The immune contexture in human tumours: impact on clinical outcome. Nat Rev Cancer. 2012;12:298–306. 22419253 10.1038/nrc3245

[39] Bruni D, Angell HK, Galon J. The immune contexture and Immunoscore in cancer prognosis and therapeutic efficacy. Nat Rev Cancer. 2020;20:662–80. 32753728 10.1038/s41568-020-0285-7

[40] Saltz J, Gupta R, Hou L, et al. Spatial Organization and Molecular Correlation of Tumor-Infiltrating Lymphocytes Using Deep Learning on Pathology Images. Cell Rep. 2018;23(1):181–e1937. 29617659 10.1016/j.celrep.2018.03.086 PMC5943714

[41] Kather JN, Pearson AT, Halama N, et al. Deep learning can predict microsatellite instability directly from histology in gastrointestinal canc*e*r. Nat Med. 2019;25:1054–6. 31160815 10.1038/s41591-019-0462-y PMC7423299

[42] Coudray N, Ocampo PS, Sakellaropoulos T, et al. Classification and mutation prediction from non–small cell lung cancer histopathology images using deep learning. Nat Med. 2018;24:1559–67. 30224757 10.1038/s41591-018-0177-5 PMC9847512

[43] Li Y, Qiu C, Tu J, Geng B, Yang J, Jiang T, Cui Q. HMDD v2.0: A database for experimentally supported human microRNA and disease associations. Nucleic Acids Res. 2014;42:D1070–4. 24194601 10.1093/nar/gkt1023 PMC3964961

[44] Huang Z, Shi J, Gao Y, Cui C, Zhang S, Li J, Zhou Y, Cui Q. HMDD v3.0: A database for experimentally supported human microRNA–disease associations. Nucleic Acids Res. 2019;47:D1013–7. 30364956 10.1093/nar/gky1010 PMC6323994

[45] Tomczak K, Czerwińska P, Wiznerowicz M. The Cancer Genome Atlas (TCGA): An immeasurable source of knowledge. Contemp Oncol. 2015;1:68–77. 10.5114/wo.2014.47136 PMC4322527 25691825

[46] Lipscomb CE. Medical subject headings (MeSH). Bull Med Libr Assoc. 2000;88(3):265. 10928714 PMC35238

[47] Wang D, Wang J, Lu M, Song F, Cui Q. Inferring the human microRNA functional similarity and functional network based on microRNA-associated diseases. Bioinformatics. 2010;26:1644–50. 20439255 10.1093/bioinformatics/btq241

[48] Lou Z, Cheng Z, Li H, Teng Z, Liu Y, Tian Z. Predicting miRNA–disease associations via learning multimodal networks and fusing mixed neighborhood information. Brief Bioinform. 2022;23:bbac159. 35524503 10.1093/bib/bbac159

[49] Xu H, et al. Dual-enhanced generative model with graph attention network and contrastive learning for aspect sentiment triplet extraction. Knowl-Based Syst. 2024;301:112342.

[50] Peng W, He Z, Dai W, Lan W. MHCLMDA: Multihypergraph contrastive learning for miRNA–disease association prediction. Brief Bioinform. 2024;25:bbad524. 10.1093/bib/bbad524 PMC10796254 38243694

[51] Li Z, Bai X, Nie R, Liu Y, Zhang L, You Z. Predicting miRNA–disease associations based on spectral graph transformer with dynamic attention and regularization. IEEE J Biomed Health Inf. 2024;28:7611–22. 10.1109/JBHI.2024.3438439 39102330

[52] Li R, Ning Q, Zhao Y, Guo S, Li H, MHMDA. Similarity–Association–Similarity metapaths and heterogeneous-hyper network learning for miRNA–disease association prediction. IEEE Trans Comput Biol Bioinform. 2025;22:203–15. 40811218 10.1109/TCBBIO.2024.3518515

[53] Xu F, Wang Y, Ling Y, Zhou C, Wang H, Teschendorff AE, Zhao Y, Zhao H, He Y, Zhang G, Yang Z. dbDEMC 3.0: Functional exploration of differentially expressed miRNAs in cancers of human and model organisms. Genomics Proteom Bioinf. 2022;20:446–54. 10.1016/j.gpb.2022.04.006 PMC9801039 35643191

[54] Jiang Q, Wang Y, Hao Y, Juan L, Teng M, Zhang X, Li M, Wang G, Liu Y. miR2Disease: A manually curated database for microRNA deregulation in human disease. Nucleic Acids Res. 2009;37:D98–104. 18927107 10.1093/nar/gkn714 PMC2686559

[55] Ferlay J, Soerjomataram I, Dikshit R, Eser S, Mathers C, Rebelo M, Parkin DM, Forman DM, Bray F. Cancer incidence and mortality worldwide: Sources, methods and major patterns in GLOBOCAN 2012. Int J Cancer. 2015;136:E359–86. 25220842 10.1002/ijc.29210

[56] Kano M, Seki N, Kikkawa N, Fujimura L, Hoshino I, Akutsu Y, Chiyomaru T, Enokida H, Nakagawa M, Matsubara H. miR-145, miR-133a and miR-133b: Tumor-suppressive miRNAs target FSCN1 in esophageal squamous cell carcinoma. Int J Cancer. 2010;127:2804–14. 21351259 10.1002/ijc.25284

[57] Ren S, Tan X, Fu MZ, Ren S, Wu X, Chen T, Latham PS, Lin P, Man Y, Fu SW. Downregulation of miR-375 contributes to ERBB2-mediated VEGFA overexpression in esophageal cancer. J Cancer. 2021;12:7138. 34729115 10.7150/jca.63836 PMC8558641

[58] Georgiannos SN, Chin Aleong J, Goode AW, Sheaff M. Secondary neoplasms of the breast. Cancer. 2001;92:2259–66. 11745279 10.1002/1097-0142(20011101)92:9<2259::aid-cncr1571>3.0.co;2-o

[59] Sugita BM, Rodriguez Y, Fonseca AS, Nunes Souza E, Kallakury B, Cavalli IJ, Cavalli R. miR-150-5p overexpression in triple-negative breast cancer contributes to the in vitro aggressiveness of this breast cancer subtype. Cancers. 2022;14:2156. 35565284 10.3390/cancers14092156 PMC9104497

[60] Gravgaard KH, Lyng MB, Laenkholm AV, Søkilde R, Nielsen BS, Litman T, Ditzel HJ. The miRNA-200 family and miRNA-9 exhibit differential expression in primary versus corresponding metastatic tissue in breast cancer. Breast Cancer Res Treat. 2012;134:207–17. 22294488 10.1007/s10549-012-1969-9

[61] Tahara E, Yasui W, Ito H, Harris CC. Recent progress in carcinogenesis, progression and therapy of lung cancer: The 19th Hiroshima Cancer Seminar – The 3rd Three Universities’ Consortium International Symposium, November 2009. Jpn J Clin Oncol. 2010;40:702–8. 20338946 10.1093/jjco/hyq031

[62] Samarth N, Gulhane P, Singh S. Immunoregulatory framework and the role of miRNA in the pathogenesis of NSCLC – A systematic review. Front Oncol. 2022;12:1089320. 36620544 10.3389/fonc.2022.1089320 PMC9811680

[63] Haggar FA, Boushey RP. Colorectal cancer epidemiology: Incidence, mortality, survival and risk factors. Clin Colon Rectal Surg. 2009;22:191–7. 21037809 10.1055/s-0029-1242458 PMC2796096

[64] Ishikawa D, Takasu C, Kashihara H, Nishi M, Tokunaga T, Higashijima J, Yoshikawa K, Yasutomo K, Shimada M. The significance of microRNA-449a and its potential target HDAC1 in patients with colorectal cancer. Anticancer Res. 2019;39:2855–60. 31177123 10.21873/anticanres.13414

