# Supplementary material for: EMGMDA: a multi-modal graph neural framework for robust prediction of miRNA–disease associations
Source: BMC Genomics. 2026 Apr 14;27:501. doi: 10.1186/s12864-026-12834-4 (PMC13188236; doi:10.1186/s12864-026-12834-4)
Supplement: Supplementary file 1 — Supplementary Material 1. [file 12864_2026_12834_MOESM1_ESM.docx]

1. **Hyperparameter settings forbaseline methods**

All parameters of the baseline method were set under the recommendations in the paper or adjusted appropriately to achieve the best performance. For convenience, we list the parameters of every baseline as follows：

(1) MINIMDA

- Optimizer: Adam
- Learning rate (lr): 0.001
- Weight decay: 0.001
- Loss function: MSE
- Epochs: 300
- Number of hidden layers (l): 2 (best performance achieved at *l = 2*)
- MLP hidden dimension: 64

(2) MDformer

- Dropout: 0.5 (HMDD v2.0), 0.3 (HMDD v3.2)
- Learning rate (lr): 1e-4
- Epochs: 40
- Number of attention heads: 4
- Projection dimension: 512
- Embedding dimension: 256

(3) MHCLMDA

- Optimizer: Adam
- Learning rate (lr): 0.002
- Contrastive temperature (τ): 0.7
- Embedding dimension: 512
- Number of hidden layers (l): 2
- Number of neighbors (K): 10
- Hyperparameter (λ):
  - 0.6 (zeroing cross-validation)
  - 0.9 (multi-column & multi-row zeroing cross-validation)

(4) DARSFormer

- Learning rate (lr): 1e-2
- Regularization coefficient (λ_reg): 1e-3
- Number of layers: 2
- Feature dropout (dropout.feat): 0.5
- Transformer dropout (dropout.trans): 0.1
- Embedding dimension: 128
- Number of attention heads: 4
- Eigenvalue encoding coefficient (λ): 100

(5) MHMDA

- Optimizer: Adam
- HeteroHyperNet layer depth: 2
- Virtual nodes ratio: 1/4
- Number of attention heads: 2
- Learning rate (lr): 0.001

1. **Statistical significance analysis of EMGMDA performance gains**

**Table S1. Paired significance tests on AUC in the HMDD v2.0 dataset**

| **Baseline** | **Mean Δ (EMGMDA − Baseline)** | **p (raw)** | **p (Holm)** | **Sig.** |
| --- | --- | --- | --- | --- |
| MINIMDA | 0.0382 | 7.24e-05 | 3.62e-04 | *** |
| MDFormer | 0.0272 | 6.56e-04 | 1.97e-03 | ** |
| MHCLMDA | 0.0242 | 2.33e-03 | 4.66e-03 | ** |
| DARSFormer | 0.0223 | 4.66e-04 | 1.86e-03 | ** |
| MHMDA | 0.0183 | 3.68e-03 | 4.66e-03 | ** |

Note: Significance markers are based on Holm–Bonferroni adjusted p-values: *** $p$ <0.001,** $p$<0.01, * $p$<0.05.

**Table S2. Paired significance tests on AUC in the HMDD v3.2 dataset**

| **Baseline** | **Mean Δ (EMGMDA − Baseline)** | **p (raw)** | **p (Holm)** | **Sig.** |
| --- | --- | --- | --- | --- |
| MINIMDA | 0.0295 | 1.29e-05 | 6.46e-05 | *** |
| MDFormer | 0.0236 | 2.56e-05 | 1.02e-04 | *** |
| MHCLMDA | 0.0287 | 1.45e-04 | 2.23e-04 | *** |
| DARSFormer | 0.0214 | 1.12e-04 | 2.23e-04 | *** |
| MHMDA | 0.0183 | 5.94e-05 | 1.78e-04 | *** |

Note: Significance markers are based on Holm–Bonferroni adjusted p-values: *** $p$ <0.001,** $p$<0.01, * $p$<0.05.

**Table S3. Paired significance tests on AUPRC in the HMDD v2.0 dataset**

| **Baseline** | **Mean Δ (EMGMDA − Baseline)** | **p (raw)** | **p (Holm)** | **Sig.** |
| --- | --- | --- | --- | --- |
| MINIMDA | 0.04108 | 4.87e-04 | 2.44e-03 | ** |
| MDFormer | 0.02600 | 1.34e-03 | 4.03e-03 | ** |
| MHCLMDA | 0.02428 | 8.99e-04 | 3.60e-03 | ** |
| DARSFormer | 0.02098 | 1.64e-03 | 4.03e-03 | ** |
| MHMDA | 0.01838 | 3.26e-03 | 4.03e-03 | ** |

Note: Significance markers are based on Holm–Bonferroni adjusted p-values: *** $p$ <0.001,** $p$<0.01, * $p$<0.05.

**Table S4. Paired significance tests on AUPRC in the HMDD v3.2 dataset**

| **Baseline** | **Mean Δ (EMGMDA − Baseline)** | **p (raw)** | **p (Holm)** | **Sig.** |
| --- | --- | --- | --- | --- |
| MINIMDA | 0.01726 | 8.19e-04 | 1.32e-03 | ** |
| MDFormer | 0.02266 | 1.83e-04 | 7.32e-04 | *** |
| MHCLMDA | 0.02636 | 4.41e-04 | 1.32e-03 | ** |
| DARSFormer | 0.02296 | 1.45e-04 | 7.23e-04 | *** |
| MHMDA | 0.01856 | 4.70e-04 | 1.32e-03 | ** |

Note: Significance markers are based on Holm–Bonferroni adjusted p-values: *** $p$ <0.001,** $p$<0.01, * $p$<0.05.

1. **Cold-start evaluation**

**Table S5.** Performance comparison under disease cold-start on HMDD v2.0

| **Methods** | **AUC** | **AUPRC** | **ACC** | **F1-score** | **Recall** | **Precision** |
| --- | --- | --- | --- | --- | --- | --- |
| MINIMDA | 0.8712 | 0.8625 | 0.7824 | 0.7881 | 0.8019 | 0.7749 |
| MDFormer | 0.8836 | 0.8789 | 0.8017 | 0.8042 | 0.8123 | 0.7963 |
| MHCLMDA | 0.8869 | 0.8814 | 0.8085 | 0.8091 | 0.8168 | 0.8016 |
| DARSFormer | 0.8897 | 0.8856 | 0.8129 | 0.8145 | 0.8237 | 0.8055 |
| MHMDA | 0.8938 | 0.8892 | 0.8184 | 0.8191 | 0.8269 | 0.8115 |
| **EMGMDA** | **0.9154** | **0.9087** | **0.8436** | **0.8469** | **0.8648** | **0.8297** |

**Table S6.** Performance comparison under miRNA cold-start on HMDD v3.2

| **Methods** | **AUC** | **AUPRC** | **ACC** | **F1-score** | **Recall** | **Precision** |
| --- | --- | --- | --- | --- | --- | --- |
| MINIMDA | 0.8926 | 0.9018 | 0.8013 | 0.8137 | 0.8214 | 0.8062 |
| MDFormer | 0.9015 | 0.8982 | 0.8276 | 0.8289 | 0.8297 | 0.8282 |
| MHCLMDA | 0.8993 | 0.8967 | 0.8239 | 0.8214 | 0.8263 | 0.8166 |
| DARSFormer | 0.9058 | 0.9016 | 0.8321 | 0.8347 | 0.8412 | 0.8284 |
| MHMDA | 0.9087 | 0.9045 | 0.8369 | 0.8385 | 0.8437 | 0.8333 |
| **EMGMDA** | **0.9289** | **0.9234** | **0.8642** | **0.8678** | **0.8796** | **0.8564** |

1. **Case-study results: top verified miRNAs and controls for histopathological image features**

**Table S7.** The top 50 verified miRNAs associated with Esophageal Neoplasms

| Esophageal Neoplasms | | |
| --- | --- | --- |
| Rank | miRNA | Reference |
| 1 | hsa-mir-125a | dbDEMC |
| 2 | hsa-mir-196a | dbDEMC |
| 3 | hsa-mir-499a | dbDEMC |
| 4 | hsa-mir-198 | dbDEMC |
| 5 | hsa-mir-29a | dbDEMC |
| 6 | hsa-mir-29b | dbDEMC |
| 7 | hsa-let-7a | dbDEMC |
| 8 | hsa-mir-141 | dbDEMC |
| 9 | hsa-mir-143 | dbDEMC |
| 10 | hsa-mir-145 | dbDEMC |
| 11 | hsa-mir-150 | dbDEMC |
| 12 | hsa-mir-15a | dbDEMC |
| 13 | hsa-mir-16 | dbDEMC |
| 14 | hsa-mir-21 | dbDEMC |
| 15 | hsa-mir-1 | dbDEMC |
| 16 | hsa-mir-133a | dbDEMC |
| 17 | hsa-mir-133b | dbDEMC |
| 18 | hsa-mir-146a | dbDEMC |
| 19 | hsa-mir-155 | dbDEMC |
| 20 | hsa-mir-208b | dbDEMC |
| 21 | hsa-mir-103a | dbDEMC |
| 22 | hsa-mir-106a | dbDEMC |
| 23 | hsa-mir-10b | dbDEMC |
| 24 | hsa-mir-126 | dbDEMC |
| 25 | hsa-mir-135a | dbDEMC |
| 26 | hsa-mir-151a | dbDEMC |
| 27 | hsa-mir-152 | dbDEMC |
| 28 | hsa-mir-17 | dbDEMC |
| 29 | hsa-mir-181b | dbDEMC |
| 30 | hsa-mir-182 | dbDEMC |
| 31 | hsa-mir-183 | dbDEMC |
| 32 | hsa-mir-191 | dbDEMC |
| 33 | hsa-mir-195 | dbDEMC |
| 34 | hsa-mir-200c | dbDEMC |
| 35 | hsa-mir-203 | dbDEMC |
| 36 | hsa-mir-204 | dbDEMC |
| 37 | hsa-mir-205 | dbDEMC |
| 38 | hsa-mir-210 | dbDEMC |
| 39 | hsa-mir-215 | dbDEMC |
| 40 | hsa-mir-221 | dbDEMC |
| 41 | hsa-mir-223 | dbDEMC |
| 42 | hsa-mir-25 | dbDEMC |
| 43 | hsa-mir-26b | dbDEMC |
| 44 | hsa-mir-31 | dbDEMC |
| 45 | hsa-mir-25 | dbDEMC |
| 46 | hsa-mir-26b | dbDEMC |
| 47 | hsa-mir-31 | dbDEMC |
| 48 | hsa-mir-34b | dbDEMC |
| 49 | hsa-mir-429 | dbDEMC |
| 50 | hsa-mir-449a | dbDEMC |

**Table S8.** The top 50 verified miRNAs associated with Breast Neoplasms

| Breast Neoplasms | | |
| --- | --- | --- |
| Rank | miRNA | Reference |
| 1 | hsa-mir-103a | dbDEMC |
| 2 | hsa-mir-106a | dbDEMC |
| 3 | hsa-mir-10b | dbDEMC |
| 4 | hsa-mir-126 | dbDEMC |
| 5 | hsa-mir-135a | dbDEMC |
| 6 | hsa-mir-151a | dbDEMC |
| 7 | hsa-mir-152 | dbDEMC |
| 8 | hsa-mir-17 | dbDEMC |
| 9 | hsa-mir-181b | dbDEMC |
| 10 | hsa-mir-182 | dbDEMC |
| 11 | hsa-mir-183 | dbDEMC |
| 12 | hsa-mir-191 | dbDEMC |
| 13 | hsa-mir-192 | dbDEMC |
| 14 | hsa-mir-193b | dbDEMC |
| 15 | hsa-mir-194 | dbDEMC |
| 16 | hsa-mir-195 | dbDEMC |
| 17 | hsa-mir-200a | dbDEMC |
| 18 | hsa-mir-200b | dbDEMC |
| 19 | hsa-mir-200c | dbDEMC |
| 20 | hsa-mir-203 | dbDEMC |
| 21 | hsa-mir-125a | dbDEMC |
| 22 | hsa-mir-196a | dbDEMC |
| 23 | hsa-mir-499a | dbDEMC |
| 24 | hsa-mir-198 | dbDEMC |
| 25 | hsa-mir-29a | dbDEMC |
| 26 | hsa-mir-29b | dbDEMC |
| 27 | hsa-let-7a | dbDEMC |
| 28 | hsa-mir-141 | dbDEMC |
| 29 | hsa-mir-143 | dbDEMC |
| 30 | hsa-mir-145 | dbDEMC |
| 31 | hsa-mir-150 | dbDEMC |
| 32 | hsa-mir-15a | dbDEMC |
| 33 | hsa-mir-16 | dbDEMC |
| 34 | hsa-mir-21 | dbDEMC |
| 35 | hsa-mir-1 | dbDEMC |
| 36 | hsa-mir-133a | dbDEMC |
| 37 | hsa-mir-133b | dbDEMC |
| 38 | hsa-mir-146a | dbDEMC |
| 39 | hsa-mir-155 | dbDEMC |
| 40 | hsa-mir-208b | dbDEMC |
| 41 | hsa-mir-204 | dbDEMC |
| 42 | hsa-mir-205 | dbDEMC |
| 43 | hsa-mir-20a | dbDEMC |
| 44 | hsa-mir-210 | dbDEMC |
| 45 | hsa-mir-215 | dbDEMC |
| 46 | hsa-mir-221 | dbDEMC |
| 47 | hsa-mir-223 | dbDEMC |
| 48 | hsa-mir-25 | dbDEMC |
| 49 | hsa-mir-26b | dbDEMC |
| 50 | hsa-mir-31 | dbDEMC |

**Table S9.** The top 50 verified miRNAs associated with Lung Neoplasms

| Lung Neoplasms | | |
| --- | --- | --- |
| Rank | miRNA | Reference |
| 1 | hsa-let-7a | dbDEMC |
| 2 | hsa-mir-141 | dbDEMC |
| 3 | hsa-mir-143 | dbDEMC |
| 4 | hsa-mir-145 | dbDEMC |
| 5 | hsa-mir-150 | dbDEMC |
| 6 | hsa-mir-15a | dbDEMC |
| 7 | hsa-mir-125a | dbDEMC |
| 8 | hsa-mir-196a | dbDEMC |
| 9 | hsa-mir-499a | dbDEMC |
| 10 | hsa-mir-198 | dbDEMC |
| 11 | hsa-mir-29a | dbDEMC |
| 12 | hsa-mir-29b | dbDEMC |
| 13 | hsa-mir-16 | dbDEMC |
| 14 | hsa-mir-21 | dbDEMC |
| 15 | hsa-mir-1 | dbDEMC |
| 16 | hsa-mir-146a | dbDEMC |
| 17 | hsa-mir-106a | dbDEMC |
| 18 | hsa-mir-10b | dbDEMC |
| 19 | hsa-mir-135a | dbDEMC |
| 20 | hsa-mir-151a | dbDEMC |
| 21 | hsa-mir-204 | dbDEMC |
| 22 | hsa-mir-205 | dbDEMC |
| 23 | hsa-mir-20a | dbDEMC |
| 24 | hsa-mir-210 | dbDEMC |
| 25 | hsa-mir-215 | dbDEMC |
| 26 | hsa-mir-221 | dbDEMC |
| 27 | hsa-mir-223 | dbDEMC |
| 28 | hsa-mir-25 | dbDEMC |
| 29 | hsa-mir-26b | dbDEMC |
| 30 | hsa-mir-31 | dbDEMC |
| 31 | hsa-mir-25 | dbDEMC |
| 32 | hsa-mir-26b | dbDEMC |
| 33 | hsa-mir-31 | dbDEMC |
| 34 | hsa-mir-34b | dbDEMC |
| 35 | hsa-mir-429 | dbDEMC |
| 36 | hsa-mir-449a | dbDEMC |
| 37 | hsa-mir-449b | dbDEMC |
| 38 | hsa-mir-92a | dbDEMC |
| 39 | hsa-mir-93 | dbDEMC |
| 40 | hsa-mir-95 | dbDEMC |
| 41 | hsa-mir-103a | dbDEMC |
| 42 | hsa-mir-106a | dbDEMC |
| 43 | hsa-mir-10b | dbDEMC |
| 44 | hsa-mir-126 | dbDEMC |
| 45 | hsa-mir-135a | dbDEMC |
| 46 | hsa-mir-151a | dbDEMC |
| 47 | hsa-mir-152 | dbDEMC |
| 48 | hsa-mir-17 | dbDEMC |
| 49 | hsa-mir-181b | dbDEMC |
| 50 | hsa-mir-182 | dbDEMC |

**Table S10.** The top 50 verified miRNAs associated with Colorectal Neoplasms

| Colorectal Neoplasms | | |
| --- | --- | --- |
| Rank | miRNA | Reference |
| 1 | hsa-mir-145 | dbDEMC |
| 2 | hsa-mir-150 | dbDEMC |
| 3 | hsa-mir-15a | dbDEMC |
| 4 | hsa-mir-16 | dbDEMC |
| 5 | hsa-mir-21 | dbDEMC |
| 6 | hsa-mir-1 | dbDEMC |
| 7 | hsa-mir-133a | dbDEMC |
| 8 | hsa-mir-103a | dbDEMC |
| 9 | hsa-mir-106a | dbDEMC |
| 10 | hsa-mir-126 | dbDEMC |
| 11 | hsa-mir-125a | dbDEMC |
| 12 | hsa-mir-196a | dbDEMC |
| 13 | hsa-mir-499a | dbDEMC |
| 14 | hsa-mir-198 | dbDEMC |
| 15 | hsa-mir-29a | dbDEMC |
| 16 | hsa-mir-29b | dbDEMC |
| 17 | hsa-let-7a | dbDEMC |
| 18 | hsa-mir-141 | dbDEMC |
| 19 | hsa-mir-143 | dbDEMC |
| 20 | hsa-mir-135a | dbDEMC |
| 21 | hsa-mir-151a | dbDEMC |
| 22 | hsa-mir-152 | dbDEMC |
| 23 | hsa-mir-17 | dbDEMC |
| 24 | hsa-mir-181b | dbDEMC |
| 25 | hsa-mir-182 | dbDEMC |
| 26 | hsa-mir-200c | dbDEMC |
| 27 | hsa-mir-204 | dbDEMC |
| 28 | hsa-mir-205 | dbDEMC |
| 29 | hsa-mir-20a | dbDEMC |
| 30 | hsa-mir-210 | dbDEMC |
| 31 | hsa-mir-215 | dbDEMC |
| 32 | hsa-mir-135a | dbDEMC |
| 33 | hsa-mir-151a | dbDEMC |
| 34 | hsa-mir-25 | dbDEMC |
| 35 | hsa-mir-26b | dbDEMC |
| 36 | hsa-mir-181b | dbDEMC |
| 37 | hsa-mir-182 | dbDEMC |
| 38 | hsa-mir-429 | dbDEMC |
| 39 | hsa-mir-449a | literature |
| 40 | hsa-mir-449b | dbDEMC |
| 41 | hsa-mir-92a | dbDEMC |
| 42 | hsa-mir-93 | dbDEMC |
| 43 | hsa-mir-95 | dbDEMC |
| 44 | hsa-mir-99b | dbDEMC |
| 45 | hsa-mir-221 | dbDEMC |
| 46 | hsa-mir-223 | dbDEMC |
| 47 | hsa-mir-1180 | dbDEMC |
| 48 | hsa-mir-1184 | dbDEMC |
| 49 | hsa-mir-31 | dbDEMC |
| 50 | hsa-mir-34b | dbDEMC |

**Table S11.** Case-study controls to isolate the contribution of histopathological image features

| **Cancer Type** | **EMGMDA (Full)** | **EMGMDA-nim (No images)** | **Image-control (Permuted)** | **Marginal Gain (Full vs. No images)** |
| --- | --- | --- | --- | --- |
| Esophageal | 50 / 50 | 47 / 50 | 46 / 50 | +3 |
| Breast | 50 / 50 | 49 / 50 | 47 / 50 | +1 |
| Lung | 50 / 50 | 48 / 50 | 46 / 50 | +2 |
| Colorectal | 49 / 50 | 46 / 50 | 45 / 50 | +3 |
| **Average Success Rate** | **99.5%** | **95.0%** | **92.0%** | **+4.5%** |
